# Cost-effectiveness of spinal manipulation, exercise, and self-management for spinal pain

**DOI:** 10.1186/s12998-025-00599-8

**Published:** 2025-08-23

**Authors:** Brent D. Leininger, Karen M. Kuntz, James S. Hodges, Roni Evans, Eva Enns, Pamela Jo Johnson, Gert Bronfort

**Affiliations:** 1 Integrative Health and Wellbeing Research Program Earl E. Bakken Center for Spirituality & Healing, University of Minnesota, Mayo Memorial Building C504, 420 Delaware Street, Minneapolis, MN 55414 USA; 2 Division of Health Policy & Management School of Public Health, University of Minnesota, 420 Delaware St SE, MMC 729 Mayo, Minneapolis, MN 55455 USA; 3 Division of Biostatistics and Health Data Science, School of Public Health, University of Minnesota, 2221 University Ave SE, Room 200 University Office Plaza, Minneapolis, MN 55414 USA; 4 Department of Public Health, North Dakota State University, 640R Aldevron Tower, 1455 14th Ave N, Fargo, ND 58102 USA

**Keywords:** Back pain, Neck pain, Cost-effectiveness, Exercise, Spinal manipulation, Self-management

## Abstract

**Background:**

The United States spends more money on the care of back and neck pain than any other health condition. Despite this, the cost-effectiveness for many recommended treatments is unclear. Our primary objective for this project was to estimate the cost-effectiveness of spinal manipulative therapy (SMT), supervised exercise therapy (ET), and home exercise and advice (HEA) for spinal pain in the U.S.

**Methods:**

We analyzed cost and clinical outcome data from eight randomized trials conducted in the U.S. using an individual participant data meta-analysis approach. We calculated cost-effectiveness from the societal and healthcare perspective of various comparisons between SMT, ET, and HEA. Incremental cost-effectiveness ratios (ICERs) were calculated using quality-adjusted life years as the main outcome.

**Results:**

The trials included a total of 1803 participants and 1488 (83%) provided complete data. Incremental cost-effectiveness ratios and probabilities of cost-effectiveness varied substantially between studies; thus, we did not conduct meta-analysis and report findings from individual trials. Cost-effectiveness findings were favorable for SMT compared to HEA for acute neck pain (ICERs below $50k/QALY) and when added to HEA for chronic back-related leg pain and chronic neck pain in older adults (better outcomes and lower costs). However, SMT was not likely cost-effective compared to HEA for chronic back pain in adults or when added to HEA for older adults (higher costs and worse outcomes). Findings for SMT were favorable when compared to ET in adults with chronic back pain and when added to ET for chronic neck pain in adults (better outcomes and lower costs) and chronic back pain in adolescents (ICERs below $50k/QALY). However, SMT is not likely cost-effective when compared to ET for chronic neck pain in adults (ICERs below $70k/QALY for exercise) and findings were inconsistent across outcomes in older adults with chronic back pain. Finally, ET may be cost-effective compared to HEA for adults with chronic neck pain (ICERs largely between $100-$200k/QALY), but not for chronic back pain or when added to HEA for older adults with chronic neck or back pain (higher costs and worse outcomes).

**Discussion:**

Cost-effectiveness findings differed between populations based on pain location, duration, and age.

**Supplementary Information:**

The online version contains supplementary material available at 10.1186/s12998-025-00599-8.

## Introduction

Spine pain (neck or back pain) is the most common chronic pain condition in the U.S. impacting nearly one in three adults over their lifetime [1, 2]. In 2016, the U.S. spent an estimated $134.5 billion in healthcare expenditures for back or neck pain, more than any other health condition [3]. Further, U.S. spending on spinal pain is increasing by $57.2 billion dollars per year, which represents one of the largest increases in healthcare spending for any condition [4]. Identifying safe and cost-effective treatment options has become a national imperative with the growing recognition that many of the current spine pain management strategies are costly, potentially harmful, and largely ineffective [5–8]. 

Conservative treatments such as spinal manipulative therapy (SMT), exercise, self-management, or other complementary and integrative interventions may reduce the clinical and cost burden of spine pain. Analyses using data from the U.S. Medical Expenditures Panel Survey and Medicare suggest conservative treatments, including SMT, can potentially reduce healthcare expenditures for spinal pain conditions; however, the cost-effectiveness of these approaches within U.S. healthcare settings has not received much attention [9–12]. Given the increasing financial and societal burden of spinal pain, and concerns surrounding current management strategies, robust cost-effectiveness analyses (CEAs) of conservative treatments for spine pain are much needed. Accordingly, there has been a call for robust CEAs of SMT and other conservative treatments for spine pain [12–14]. 

This project’s purpose was to estimate the incremental cost-effectiveness of spinal manipulative therapy, exercise therapy, and self-management for spinal pain in U.S. settings from both societal and healthcare perspectives using quality-adjusted life years (QALYs), pain intensity, and disability as effectiveness measures.

## Methods

We analyzed cost and clinical outcome data collected as part of eight randomized clinical trials performed in the U.S. using an individual participant data meta-analysis (IPDMA) approach. An IPDMA approach has many advantages over traditional meta-analysis including the ability to conduct standardized within-study analyses, account for missing data at the individual level, and investigate potential sub-group effects at the participant level which may account for heterogeneity in estimates across studies [15]. 

The eight randomized clinical trials were conducted by our research group and used similar methods, collected similar clinical outcome, healthcare utilization, and work productivity data, and included different combinations of SMT, exercise therapy, or self-management for spinal pain. Combined analyses of economic data are rarely possible due to differences in resource utilization outcomes, costs, and healthcare settings [16]. Also, individual clinical trials rarely include enough participants to detect important differences in economic outcomes. This project represented a unique opportunity to potentially combine clinical and economic data collected in eight randomized clinical trials using an IPDMA approach as the protocols had substantial overlap of assessed treatments and consistency in treatment and data collection and all of the studies were conducted in the United States. All of the included trials were designed with a blend of pragmatic and explanatory features based on the PRECIS-2 tool [17]. Common pragmatic features included broad inclusion and narrow exclusion criteria, routine follow-up for adherence to study interventions, a primary outcome important to patients (pain severity), and intention-to-treat primary analyses. Common explanatory features included multi-faceted recruitment strategies to facilitate timely and representative enrollment, the use of University-affiliated research clinicians to deliver the interventions, and detailed follow-up to fully assess the impact of the interventions. Many of the explanatory features were chosen to ensure the trials were successful (completed on time with representative population and high data collection) and the interventions were applied with fidelity when assessing effectiveness.

Each trial obtained written consent from participants who were 18 years of age or older, and written patient assent and parent consent from participants 12–17 years of age. Six of the trials were funded by the U.S. Department of Health and Human Services Health Resources and Services Administration [18–23] and one was funded by the National Institute of Health’s National Center for Complementary and Integrative Health [24]. Seven of the trials are registered on clinicaltrials.gov (one trial [25] was initiated before its existence). A protocol outlining our detailed methods has been published [26]. Table 1 provides an overview of trial populations and interventions.

**Table 1:** Clinical trial populations and interventions

Clinicaltrials.gov ID#	Population			Interventions		
	Condition	Sample	Age	Group 1	Group 2	Group 3
Pre-dates ^24^	Chronic neck pain	191	22–66	SMT + ET	ET	SMT
NCT00269360^19^	Chronic neck pain	270	20–65	SMT + ET	ET	HEA
NCT00269308^21^	Chronic neck pain	241	65–88	SMT + HEA	ET + HEA	HEA
NCT00029770^23^	Acute neck pain	272	21–85	SMT	HEA	Medication
NCT00269347^18^	Chronic low back pain	301	22–66	SMT	ET	HEA
NCT00269321^22^	Chronic low back pain	240	65–91	SMT + HEA	ET + HEA	HEA
NCT00494065^17^	Chronic back-related leg pain	192	27–92	SMT + HEA	HEA	-
NCT01096628^20^	Chronic low back pain	184	12–18	SMT + ET	ET	-

CNP=Chronic neck pain; ANP=Acute neck pain; CLBP=Chronic low back pain; BRLP=Back related leg pain; SMT=Spinal manipulative therapy; ET=Supervised exercise therapy; HEA=Home exercise and advice

### Settings and participants

All the clinical trials were performed within a university-affiliated research clinic in the Minneapolis, MN metropolitan region. Six of the clinical trials were performed exclusively in MN [19, 20, 22–25] while two were multi-center studies with additional sites in Portland, OR [21] or Davenport, IA [18]. Participants had commonly recognized sub-groups of spinal pain including acute or chronic neck pain, chronic low back pain, and back-related leg pain (Table 1). Five of the trials included adults (18–65 years), two trials included older adults (65 years and older), and one trial focused on adolescents (12–18 years). All eight trials recruited participants from the general population primarily through mass mailings. Other recruitment strategies included advertisement in newspapers, social media, television, radio, and community posters.

## Inclusion/Exclusion criteria

Randomized clinical trials for back or neck pain conditions were included if they were conducted in the U.S., compared spinal manipulation, exercise therapy, or self-management interventions to one another, collected economic outcomes, and provided access to individual level data. The eight clinical trials conducted by our research group met these criteria and were included in the project. A literature search in Pubmed identified a number of additional trials assessing the cost-effectiveness of SMT, exercise therapy, or self-management [11, 12, 27–39]. Data from these trials was not sought, due to differences in treatment comparisons and healthcare settings (i.e., studies were either not conducted in the U.S [27–37]. or did not compare spinal manipulation, exercise therapy, or self-management interventions to one another [38]). In all eight included trials, participants were required to have self-reported spinal pain severity ≥ 3/10 for inclusion. Other common inclusion criteria were stable medication plan and no ongoing spinal treatment prior to enrollment. Common exclusion criteria included current or pending litigation, inability to read and comprehend English, substance abuse, history of surgical spinal fusion, progressive neurological deficits, or contraindications to study treatments.

## Interventions

Spinal manipulative therapy was included as an intervention in all eight studies. Supervised exercise therapy [19–23, 25] and self-management (home exercises and advice) [18–23] was also included in six of the trials. Trial interventions were provided to study participants at no cost.

**Spinal manipulative therapy (SMT)** was delivered by licensed chiropractors over a 12-week intervention period in all studies. The treating chiropractor determined the frequency of SMT visits in six of the eight studies. The mean frequency of SMT visits ranged from 10 to 20 with most trials reporting a mean visit frequency between 15 and 20. SMT consisted of high velocity, low amplitude manipulation, with an option to use low velocity, variable amplitude mobilization as indicated. Brief soft tissue work (up to five minutes) and heat therapy were allowed to facilitate the manual treatment, if necessary, which is typical in clinical practice.

**Supervised exercise therapy (ET)** was delivered by licensed chiropractors, physical therapists, or exercise therapists over a 12-week period in six trials. Five studies [19, 20, 22, 23, 25] tested 20 one-hour visits of one-on-one supervised exercise therapy and one trial [21] assessed 8 to 16, 45-minute visits with the number of visits individualized based on patient response and needs. Participants completed a combination of stretching and strengthening exercises emphasizing a high number of repetitions with progressions in challenge and/or resistance over time. Exercises were tailored for each participant’s abilities and could include the use of labile surfaces in addition to balance and coordination exercises. Participants also completed a light aerobic warm-up (up to 10 min) in all the trials.

**Home exercise and advice (HEA)** was delivered by licensed chiropractors or exercise therapists in six trials [18–23]. Participants attended two to four one-hour visits where they were given information on their prognosis, self-care advice, and home exercise instruction. Home exercises typically included a combination of self-mobilization, stretching and strengthening exercises specific to the individual’s condition and ability.

**Intervention Combinations** Each trial included SMT, ET, or HEA either alone or in combination with one of the other treatments. Each trial included and compared a maximum of three treatment approaches. No trial assessed all possible treatment strategies (See Table 1). We compared the following treatment approaches which were assessed in more than a single trial:

1. SMT vs. HEA
2. SMT + HEA vs. HEA
3. ET vs. SMT
4. SMT + ET vs. ET
5. ET vs. HEA
6. ET + HEA vs. HEA

The comparison of SMT to medication was only present in a single trial and was not included in the analysis.

## Perspective, time horizon & discount rate

We adopted a societal perspective for the primary analysis including all healthcare costs regardless of payer (third-party insurers, patient out-of-pocket costs) in addition to time and transportation costs associated with healthcare use and lost productivity costs for both paid and unpaid labor related to spinal pain. We excluded future earnings and consumption costs since interventions for spinal pain are not expected to impact mortality. In addition to the societal perspective, we adopted a healthcare perspective including only healthcare costs [40]. Table 2 provides a summary of resources included in the healthcare and societal perspectives. All eight trials collected clinical outcome and healthcare utilization data for one year, which was the time horizon for the cost-effectiveness evaluation. No discounting (diminishing future costs and health effects to represent present value) was applied due to the limited one-year time horizon.

**Table 2:** Cost components included in the societal and healthcare perspectives

Cost component	Perspective	
	Healthcare	Societal
**Formal healthcare sector**		
Paid for by third-party payers	X	X
Paid for by patients	X	X
**Informal healthcare sector**		
Patient time	-	X
Transportation costs	-	X
**Non-healthcare sector**		
Productivity costs (paid and unpaid labor)	-	X

## Outcomes

Clinical and cost outcomes were collected by self-report at multiple time points over a one-year period with similar timing (4, 12, 26, and 52 weeks) across studies. Clinical outcomes included pain, disability, quality of life, and work absenteeism.

### Clinical outcomes

QALYs (a metric combining quality and quantity of life) were constructed using the SF6D, collected in seven trials, and the EQ5D, collected in six trials. We used U.S. preferences for individual SF6D health states obtained via discrete choice experimentation [41]. QALYs were also estimated using U.S. preferences for health states in the EuroQol 5D-3L (EQ5D) as a sensitivity analysis [42]. Finally, one study of adolescents assessed health related quality of life using the pediatric quality of life inventory (PedsQL) which has recently been mapped to the EQ5D in a UK adolescent population [43]. 

Self-reported pain intensity was measured using the 11-box numerical rating scale (NRS) and was the primary outcome in each of the eight trials. The NRS is a reliable and valid outcome measure for individuals with spinal pain and is recommended as a core outcome domain by both the Initiative on Methods, Measurement, and Pain Assessment in Clinical Trials group and the NIH task force on research standards for chronic low back pain [44, 45]. 

Disability was measured with reliable and valid measures commonly used in spine pain research: the Neck Disability Index (four trials) [46, 47] and Roland-Morris Disability Questionnaire (four trials) [48]. Standardized mean differences were used for disability analyses to provide a uniform scale for meta-analysis of outcomes measured with different scales [49]. 

## Cost outcomes

Direct Healthcare Costs: We collected healthcare utilization outcomes including the number of provider visits by specialty, types of services provided, and medication use. The number of provider visits and medication use were collected using standardized self-report questionnaires in all eight trials, and more detailed information regarding the types of services provided (e.g., MRI, injections) was collected by phone interviews in five of the trials [18–20, 22, 23]. A list of procedures and corresponding Current Procedural Terminology (CPT)/Healthcare Common Procedure Coding System (HCPCS) codes was compiled and unit costs for each procedure were determined using Medicare’s national allowable payment (Appendix Table 1). Unit costs for non-covered services under Medicare (such as acupuncture) were determined using Medicare’s published relative value unit for the corresponding CPT code. Unit costs for medication were determined using the average cost per prescription day from Medicare’s prescription drug profiles public use file. All unit cost estimates were converted to 2020 U.S. dollars using the Centers for Medicare and Medicaid Services Personal Health Care Expenditure deflator to account for inflation [40]. 

Productivity Costs: A human capital approach was used including lost productivity costs for both paid and unpaid labor (such as retirees or homemakers) [50]. Lost work productivity due to absenteeism was collected using a modified question from the U.S. National Health Interview Survey (seven trials) [51]. Participants reported the number of days in the past month they were unable to carry out their daily work (in or away from home) due to spine pain. We valued each day as eight hours of reduced productivity using age-specific U.S. national pre-tax median hourly wage rates plus fringe benefits [40]. The trial conducted in an adolescent population did not include work absenteeism measures.

Time & Transportation Costs: Time and transportation costs associated with healthcare utilization were included using an opportunity cost approach (valuing resources according to their best alternative use). A standardized time unit for each procedure was multiplied by the age-specific U.S. national post-tax median wage rate plus fringe benefits. Healthcare-related transportation costs were estimated using average distance and transportation time estimates for medical care in the U.S. as reported in the National Household Travel Survey [52]. Transportation time was valued using age-specific national post-tax median wage rates plus fringe benefits [40]. The U.S. Internal Revenue Service’s standard mileage deduction rate for operating an automobile was used to value transportation costs.

## Analyses

### Effectiveness analyses

We determined mean clinical outcomes using time weighted averages over the one-year time horizon using linear interpolation. Differences in outcomes were analyzed using linear regression with the baseline measure of the outcome included as a covariate.

### Cost outcomes analyses

Cost data for healthcare and medication use, time and transportation, and lost productivity were analyzed using generalized linear models with a gamma distribution and identity link to model mean costs over the one year time horizon [53]. 

### Cost-effectiveness analyses

We ranked treatments by mean outcome and determined the incremental cost-effectiveness ratio (ICER) by dividing incremental costs by incremental effects. Dominant treatments were defined as having lower costs and better effectiveness on average. We did not calculate ICERs for treatments which were dominated (more expensive, less effective); however, we reported uncertainty of cost and effect differences for dominated interventions [54]. Bias-corrected bootstrap confidence intervals were calculated using 5000 samples taken with replacement with the subject as the unit of observation. Bootstrapped cost-effect pairs were plotted on the cost-effectiveness plane to graphically display uncertainty surrounding the ICER [55]. Cost-effectiveness analyses using QALYs as the effectiveness measure are sometimes referred to as cost-utility analyses [56]. Given that there are a variety of historical economic theories and definitions of “utility” and methods for constructing QALYs, we have chosen to describe these as cost-effectiveness analyses using QALYs [40, 56]. We used cost-effectiveness acceptability curves (CEACs) to determine the probability each treatment is cost-effective based on a range of recommended willingness to pay thresholds for a QALY within the U.S [57]. CEACs provide a graphical display of uncertainty that an intervention is cost-effective at different willingness to pay thresholds for one year of perfect health. We also conducted net monetary benefit analyses to display confidence intervals over a large range of willingness-to-pay thresholds (the amount of money a decision maker would be willing to pay for one year of full health given a fixed budget) [58]. 

### Missing data

Missing clinical outcome and cost data were imputed separately for each treatment group using multiple imputation (Procedure MI in STATA). For each study, ten imputed data sets were created using a multivariate normal model for clinical outcomes and predictive mean matching for costs [59]. The imputation models included clinical outcomes in addition to baseline covariates associated with missing data.

### Individual participant data Meta-analysis (IPDMA)

We used a two-stage approach for IPDMA and followed recommended guidelines for standard and IPDMA analyses [15, 60, 61]. 

Stage One: First, for each perspective (societal, healthcare) and comparison (e.g., SMT vs. HEA), we identified trials for possible meta-analysis. Next, we computed individual trial estimates for differences in effectiveness, costs, and incremental cost-effectiveness.

Stage Two: Meta-analysis of cost-effectiveness outcomes is more challenging than clinical outcomes because in addition to heterogeneity issues there are practical concerns for how ICERs are interpreted and how uncertainty is assessed [62, 63]. We considered each of these factors when deciding whether meta-analysis was appropriate. First, we visually inspected individual trial estimates of effectiveness and costs using forest plots and determined the amount of statistical heterogeneity using the I [2] statistic. Next, we inspected ICERs and cost-effectiveness acceptability curves from each individual study to ensure similar cost-effectiveness interpretation. We did not combine studies when ICERs were in different quadrants on the cost-effectiveness plane (e.g., lower costs and better outcomes in one study and higher costs and better outcomes in another). We also did not combine studies when the probabilities of cost-effectiveness on CEACs differed by more than 20% points (e.g., 50% vs. 30%). When ICERs or cost-effectiveness acceptability curves were not similar, we reported the individual trial cost-effectiveness results. If pooling was appropriate with similar cost-effectiveness interpretation from the individual trials, we planned to combine studies using random effects models.

We conducted an overall synthesis of cost-effectiveness for compared treatments based on (1) the estimated ICER for QALYs; (2) the probability of cost-effectiveness across the range of recommended willingness-to-pay (WTP) thresholds for a QALY in the U.S. where $50k/QALY is the lower boundary and $200k/QALY is the upper boundary for cost-effectiveness; [64] and (3) the consistency of findings across outcomes and perspectives. Cost-effectiveness was rated as very likely, likely, maybe/similar, not likely, and very unlikely using the following criteria:

- Very Likely = Dominant or ICERs below $50k/QALY; 80% or greater probability of cost-effectiveness at WTP thresholds above $100k/QALY; consistent findings across outcomes/perspectives;
- Likely = Dominant or ICERs below $100k/QALY; 60–80% probability of cost-effectiveness at WTP thresholds above $100k/QALY; findings mostly consistent across outcomes/perspectives;
- Maybe/Similar = ICERs between $100k and $200k/QALY; 40 to 60% probability of cost-effectiveness for WTP thresholds between $100k and $200k/QALY; inconsistency across outcomes/perspectives;
- Not Likely = Dominated or ICERs above $150k/QALY; 20 to 40% probability of cost-effectiveness for WTP thresholds between $100 and $200k/QALY; findings mostly consistent across outcomes/perspectives;
- Very Unlikely = Dominated or ICERs above $200k/QALY with less than 20% probability of cost-effectiveness for WTP thresholds below $200k/QALY; consistent findings across outcomes/perspectives.

A limited number of sub-group analyses were planned (age, pain location, pain duration), but could not be completed due to a limited number of subjects within each sub-group for each comparison.

## Results

We included data from 1803 participants across eight randomized trials. Complete clinical outcome and cost data was available for 1488 (83%) of participants. Results are presented by treatment comparison below (Tables 3, 4, 5, 6, 7 and 8). While statistical heterogeneity for effectiveness outcomes was often low, heterogeneity for cost outcomes was higher. Importantly, individual trials often differed in ICER interpretation and uncertainty analyses consistently found large differences in cost-effectiveness probability at important willingness to pay thresholds (e.g. $100,000/QALY). For example, adding SMT to HEA resulted in less costs and better outcomes for adults with back-related leg pain with a cost-effectiveness probability near 100% at $100,000/QALY, but higher costs and worse outcomes for older adults with chronic back pain with a cost-effectiveness probability near 30% at $100,000/QALY. Accordingly, we present cost-effectiveness results only for the individual trials. Figures detailing the uncertainty of ICER estimates on the cost-effectiveness plane for all outcomes, net monetary benefit findings for QALY outcomes, and cost-effectiveness acceptability curves for pain and disability reduction are displayed in the appendix.

### SMT vs. HEA

Two trials with 383 participants included data for this comparison; [19, 24] one included adults with acute neck pain and the other included adults with chronic low back pain (Table 3). Complete cost and clinical outcome data was available for 295 (77%) participants. ICERs and cost-effectiveness acceptability curves differed substantially by trial, so cost-effectiveness outcomes were not pooled (e.g., ICERs below $44k/QALY for acute neck pain, but not calculated for chronic back pain because SMT had higher costs and lower QALYs). Differences between treatments in clinical outcomes over the one-year time horizon were small and not statistically significant across studies. Clinical outcomes favored SMT over HEA for adults with acute neck pain, and HEA over SMT for adults with chronic back pain. Societal and healthcare costs were higher for SMT compared to HEA, and differences in healthcare costs were statistically significant in both trials. On average, societal costs were $611 higher for adults with acute neck pain (95% CI -$1243 to $2396) and $82 higher for adults with chronic back pain (-$2577 to $2291). Healthcare costs were $262 higher for adults with acute neck pain (95% CI $138 to $437) and $311 higher for adults with chronic back pain (95% CI $158 to $461).

**Table 3:** Cost-effectiveness results for spinal manipulative therapy relative to home exercise and advice

	# trials	# subjects	Δ Societal Costs (95% CI)†	I^2^	Δ Healthcare Costs (95% CI) †	I^2^	Δ Outcome (95%CI) †	I^2^	ICER (Societal)	ICER (Healthcare)
**QALYs (SF6D)**	2	383	$422 (-$1064 to $1836)	0%	$286 ($187 to $437)	0%	0.003 (−0.014 to 0.020)	34%	--	--
ANP	--	182	$611 (-$1243 to $2396)	--	$262 ($138 to $437)	--	0.014 (−0.011 to 0.039)	--	$43,614/QALY	$18,747/QALY
CLBP	--	201	$82 (-$2577 to $2291)	--	$311 ($158 to $461)	--	−0.008 (−0.031 to 0.017)	--	HEA Dominant‡	HEA Dominant‡
**QALYs (EQ5D)**	2	383	$422 (-$1064 to $1836)	0%	$286 ($187 to $437)	0%	−0.003 (−0.018 to 0.013)	0%	--	--
ANP	--	182	$611 (-$1243 to $2396)	--	$262 ($138 to $437)	--	0.002 (−0.018 to 0.022)	--	$305,300/QALY	$131,230/QALY
CLBP	--	201	$82 (-$2577 to $2291)	--	$311 ($158 to $461)	--	−0.010 (−0.034 to 0.013)	--	HEA Dominant‡	HEA Dominant‡
**Pain reduction**	2	383	$422 (-$1064 to $1836)	0%	$286 ($187 to $437)	0%	0.06 (−0.28 to 0.35)	0%	--	--
ANP	--	182	$611 (-$1243 to $2396)	--	$262 ($138 to $437)	--	0.15 (−0.31 to 0.62)	--	$4,070	$1,749
CLBP	--	201	$82 (-$2577 to $2291)	--	$311 ($158 to $461)	--	−0.03 (−0.52 to 0.40)	--	HEA Dominant‡	HEA Dominant‡
**Disability reduction (SMD)**	2	383	$422 (-$1064 to $1836)	0%	$286 ($187 to $437)	0%	−0.07 (−0.27 to 0.10)	0%	--	--
ANP	--	182	$611 (-$1243 to $2396)	--	$262 ($138 to $437)	--	0.03 (−0.24 to 0.29)	--	$20,354	$8,749
CLBP	--	201	$82 (-$2577 to $2291)	--	$311 ($158 to $461)	--	−0.16 (−0.44 to 0.07)	--	HEA Dominant‡	HEA Dominant‡

† Bias-corrected bootstrap confidence intervals; ‡ Dominant = lower mean costs and better mean outcomes; ICER = Incremental cost-effectiveness ratio; QALY = quality adjusted life year; ANP = acute neck pain trial in adults; CLBP = chronic low back pain trial in adults; SMD = Standardized Mean Difference

**Fig. 1:**
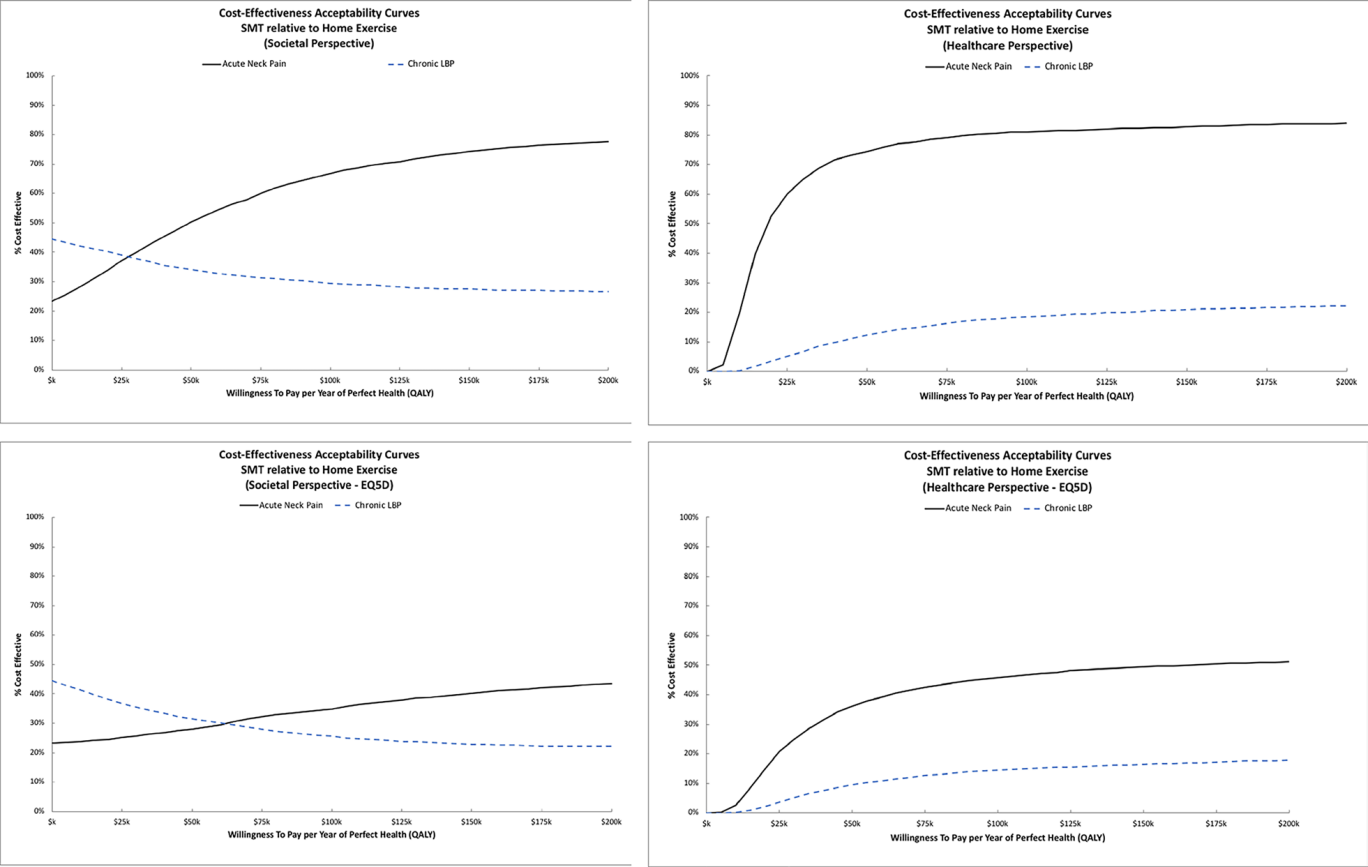
Cost-effectiveness acceptability curves for Spinal Manipulative Therapy relative to Home Exercise and Advice. The top row displays findings from the primary analysis. The bottom row displays findings from the sensitivity analysis using EQ5D for QALYs. The societal perspective is shown in the left column and the healthcare perspective is shown in the right column

The individual trial ICER estimates and cost-effectiveness acceptability curves varied substantially. For adults with acute neck pain, SMT costs more and was more effective on average. ICERs were $44k/QALY from the societal perspective and $19k/QALY from the healthcare perspective. Sensitivity analyses assessing differences in QALYs using the EQ5D instead of the SF6D resulted in smaller QALY differences (mean difference of 0.002 QALYs) and higher ICERs ($305k/QALY for societal perspective; $131k/QALY for healthcare perspective). Figure 1 shows cost-effectiveness acceptability curves for SMT relative to HEA across perspectives and QALY outcomes. For acute neck pain, the probability SMT is cost-effective relative to HEA is near 70% for the societal perspective and 80% for the healthcare perspective at WTP thresholds of $100k/QALY and higher. In the sensitivity analyses using EQ5D QALYs, the probability of cost-effectiveness decreases to roughly 40% for the societal perspective and 50% for the healthcare perspective at WTP thresholds of $100k/QALY and higher. For adults with chronic back pain, SMT cost more and was less effective than HEA across perspectives and outcomes with probabilities of cost-effectiveness that were below 50% for a wide range of WTP thresholds.

### SMT + HEA vs. HEA

Three trials with 512 participants included data for this comparison (Table 4) [18, 22, 23]. One trial included adults with back-related leg pain, the second assessed older adults with chronic low back pain, and the third included older adults with chronic neck pain. Complete cost and clinical outcome data was available for 445 (87%) participants. Cost-effectiveness outcomes were not pooled due to important differences between studies in estimated ICERs and cost-effectiveness acceptability curves (e.g., adding SMT to HEA resulted in lower costs and higher QALYs in back-related leg pain, but higher costs and lower QALYs in older adults with chronic back pain). Differences between treatments in clinical outcomes were small and generally not statistically significant across studies. Across all three studies, clinical outcomes favored adding SMT to HEA, except for QALYs for older adults with chronic low back pain. The only statistically significant finding favored adding SMT to HEA for reducing pain severity in older adults with chronic neck pain. Differences in pain and disability reduction pooled across studies were statistically significant, but small in magnitude with a mean difference in pain reduction of 4% points (0.41 on a 0–10 scale) and a standardized mean difference in disability reduction of 0.19. Differences in costs varied by population and perspective. Both societal and healthcare costs were significantly lower when adding SMT to HEA for adults with back-related leg pain (mean difference of $4918 lower for societal perspective and $1804 lower for healthcare perspective). Healthcare costs were significantly higher for older adults with chronic neck ($724 higher) and low back pain ($1022 higher). Societal costs were not significantly different in the two trials with older adults and were lower when adding SMT to HEA for chronic neck pain ($541 lower), but higher for chronic low back pain ($949 higher).

**Table 4:** Cost-effectiveness results for adding spinal manipulative therapy to home exercise and advice

	# trials	# subjects	Δ Societal Costs (95% CI)†	I^2^	Δ Healthcare Costs (95% CI) †	I^2^	Δ Outcome (95%CI) †	I^2^	ICER (Societal)	ICER (Healthcare)
**QALYs (SF6D)**	3	512	-$1382 (-$3574 to $182)	56%	$194 (-$591 to $427)	79%	0.008 (−0.006 to 0.023)	0%	--	--
BRLP	--	192	-$4918 (-$9325 to -$1312)	--	-$1804 (-$3556 to -$356)	--	0.018 (−0.004 to 0.044)	--	SMT + HEA Dominant‡	SMT + HEA Dominant‡
SLBP	--	161	$949 (-$3390 to $5032)	--	$1022 ($413 to $2369)	--	−0.008 (−0.037 to 0.019)	--	HEA Dominant‡	HEA Dominant‡
SNP	--	159	-$541 (-$3015 to $1604)	--	$724 ($324 to $1651)	--	0.009 (−0.018 to 0.034)	--	SMT + HEA Dominant‡	$80,497/QALY
**QALYs (EQ5D)**	3	512	-$1382 (-$3574 to $182)	56%	$194 (-$591 to $427)	79%	0.002 (−0.013 to 0.014)	39%	--	--
BRLP	--	192	-$4918 (-$9325 to -$1312)	--	-$1804 (-$3556 to -$356)	--	0.015 (−0.011 to 0.043)	--	SMT + HEA Dominant‡	SMT + HEA Dominant‡
SLBP	--	161	$949 (-$3390 to $5032)	--	$1022 ($413 to $2369)	--	−0.013 (−0.036 to 0.005)	--	HEA Dominant‡	HEA Dominant‡
SNP	--	159	-$541 (-$3015 to $1604)	--	$724 ($324 to $1651)	--	0.008 (−0.015 to 0.031)	--	SMT + HEA Dominant‡	$90,560/QALY
**Pain reduction**	3	512	-$1382 (-$3574 to $182)	56%	$194 (-$591 to $427)	79%	0.41 (0.15 to 0.68)	0%	--	--
BRLP	--	192	-$4918 (-$9325 to -$1312)	--	-$1804 (-$3556 to -$356)	--	0.48 (−0.002 to 0.95)	--	SMT + HEA Dominant‡	SMT + HEA Dominant‡
SLBP	--	161	$949 (-$3390 to $5032)	--	$1022 ($413 to $2369)	--	0.15 (−0.32 to 0.66)	--	$6326	$6811
SNP	--	159	-$541 (-$3015 to $1604)	--	$724 ($324 to $1651)	--	0.56 (0.12 to 1.00)	--	SMT + HEA Dominant‡	$1294
**Disability reduction (SMD)**	3	512	-$1382 (-$3574 to $182)	56%	$194 (-$591 to $427)	79%	0.19 (0.04 to 0.37)	0%	--	--
BRLP	--	192	-$4918 (-$9325 to -$1312)	--	-$1804 (-$3556 to -$356)	--	0.24 (−0.02 to 0.50)	--	SMT + HEA Dominant‡	SMT + HEA Dominant‡
SLBP	--	161	$949 (-$3390 to $5032)	--	$1022 ($413 to $2369)	--	0.09 (−0.22 to 0.40)	--	$10,544	$11,352
SNP	--	159	-$541 (-$3015 to $1604)	--	$724 ($324 to $1651)	--	0.24 (−0.05 to 0.56)	--	SMT + HEA Dominant‡	$3,018

† Bias-corrected bootstrap confidence intervals; ‡ Dominant = lower mean costs and better mean outcomes; ICER = Incremental cost-effectiveness ratio; QALY = quality adjusted life year; BRLP = back-related leg pain trial in adults; SLBP = chronic low back pain trial in older adults (seniors); SMD = Standardized Mean Difference; SNP = chronic neck pain trial in older adults (seniors)

**Fig. 2:**
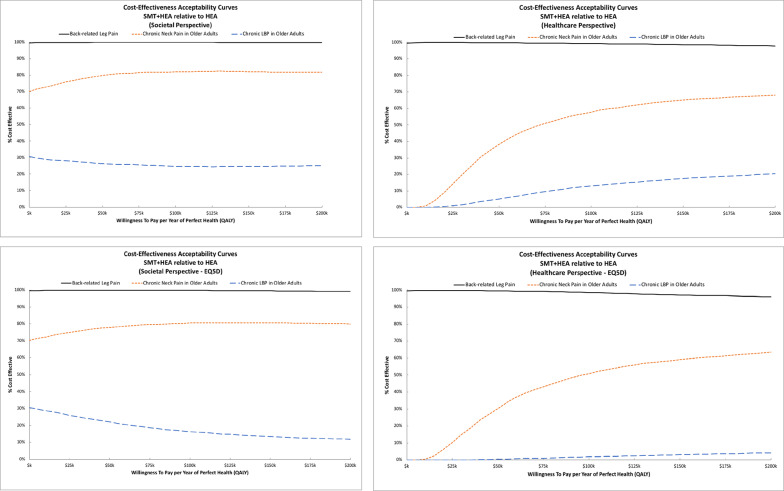
Cost-effectiveness acceptability curves for adding Spinal Manipulative Therapy to Home Exercise and Advice. The top row displays findings from the primary analysis. The bottom row displays findings from the sensitivity analysis using EQ5D for QALYs. The societal perspective is shown in the left column and the healthcare perspective is shown in the right column

ICER estimates and cost-effectiveness acceptability curves varied between trials. For adults with back-related leg pain, adding SMT to HEA costs less and was more effective across all outcomes and perspectives. For older adults with chronic neck pain, adding SMT to HEA cost less and was more effective from the societal perspective. Costs for adding SMT to HEA were higher from the healthcare perspective resulting in ICERs ranging from $80k to $91k per QALY. For older adults with chronic low back pain, adding SMT to HEA cost more and was less effective in terms of QALYs (i.e., it was dominated by HEA alone). Figure 2 shows cost-effectiveness acceptability curves for adding SMT to HEA. For adults with back-related leg pain, adding SMT to HEA has a very high probability of cost-effectiveness across perspectives and WTP thresholds. For older adults with chronic low back pain, there is a low probability (< 30%) that adding SMT to HEA is cost-effective across perspectives and WTP thresholds. For older adults with chronic neck pain, the probability that adding SMT to HEA is cost-effective is above 70% regardless of WTP threshold for the societal perspective. For the healthcare perspective, the probability of cost-effectiveness plateaus at around 60% for WTP thresholds above $100k/QALY. Sensitivity analyses using EQ5D QALYs had similar findings across all three trials.

### ET vs. SMT

Four trials with 650 participants included data for this comparison (Table 5) [19, 22, 23, 25]. Two trials focused on chronic back pain and the other two focused on neck pain. The population was limited to older adults in one each of the chronic back and chronic neck pain trials. Complete cost and clinical outcome data was available for 551 (85%) participants. Cost-effectiveness outcomes were not pooled due to heterogeneity between studies in estimated ICERs and cost-effectiveness acceptability curves (ICERs for ET relative to SMT were below $29k/QALY in two studies, but ET was dominated by SMT with higher costs and lower QALYs in two studies). Differences between treatments in clinical outcomes were small and generally not statistically significant across studies. Differences in clinical outcomes consistently favored ET for chronic neck pain, with a significant improvement in pain reduction (mean difference 0.61; 95% CI 0.01 to 1.13). For older adults with chronic neck pain, differences in clinical outcomes consistently favored SMT, but were not significant. Differences in clinical outcomes for the two chronic back pain studies did not consistently favor one treatment over the other. ET resulted in higher costs consistently across trials from both the societal and healthcare perspectives, with most differences being statistically significant. Societal costs were over $2000 higher with ET for chronic back pain and chronic neck pain in older adults. Differences in societal costs were lower and not statistically significant for chronic neck pain ($524) and chronic back pain in older adults ($433). Healthcare costs were significantly higher across trials for ET relative to SMT, with differences ranging from $1394 in older adults with chronic neck pain to $1965 for chronic back pain.

**Table 5:** Cost-effectiveness results for exercise therapy relative to spinal manipulative therapy

	# trials	# subjects	Δ Societal Costs (95% CI)†	I^2^	Δ Healthcare Costs (95% CI) †	I^2^	Δ Outcome (95%CI) †	I^2^	ICER (Societal)	ICER (Healthcare)
**QALYs (SF6D)**	4	650	$1915 ($1093 to $3225)	0%	$1746 ($1668 to $1999)	73%	0.004 (−0.011 to 0.016)	12%	--	--
CLBP	--	200	$2501 ($388 to $4551)	--	$1965 ($1788 to $2127)	--	−0.001 (−0.028 to 0.022)	--	SMT Dominant‡	SMT Dominant‡
CNP I	--	127	$524 (-$2164 to $2966)	--	$1647 ($1548 to $1756)	--	0.024 (−0.010 to 0.058)	--	$21,827/QALY	$68,643/QALY
SLBP	--	161	$433 (-$3192 to $4191)	--	$1769 ($317 to $3358)	--	0.015 (−0.013 to 0.044)	--	$28,849/QALY	$117,926/QALY
SNP	--	162	$2235 ($771 to $3458)	--	$1394 ($573 to $1816)	--	−0.010 (−0.033 to 0.013)	--	SMT Dominant‡	SMT Dominant‡
**QALYs (EQ5D)**	3	523							--	--
CLBP	--	200	$2501 ($388 to $4551)	--	$1965 ($1788 to $2127)	--	0.016 (−0.011 to 0.042)	--	$156,310/QALY	$122,825/QALY
SLBP	--	161	$433 (-$3192 to $4191)	--	$1769 ($317 to $3358)	--	0.019 (−0.005 to 0.045)	--	$22,776/QALY	$93,100/QALY
SNP	--	162	$2235 ($771 to $3458)	--	$1394 ($573 to $1816)	--	−0.015 (−0.037 to 0.008)	--	SMT Dominant‡	SMT Dominant‡
**Pain reduction**	4	650	$1915 ($1093 to $3225)	0%	$1746 ($1668 to $1999)	73%	0.05 (−0.20 to 0.29)	68%	--	--
CLBP	--	200	$2501 ($388 to $4551)	--	$1965 ($1788 to $2127)	--	0.26 (−0.19 to 0.70)	--	$9,619	$7,558
CNP I	--	127	$524 (-$2164 to $2966)	--	$1647 ($1548 to $1756)	--	0.61 (0.01 to 1.13)	--	$859	$2,701
SLBP	--	161	$433 (-$3192 to $4191)	--	$1769 ($317 to $3358)	--	−0.19 (−0.67 to 0.30)	--	SMT Dominant‡	SMT Dominant‡
SNP	--	162	$2235 ($771 to $3458)	--	$1394 ($573 to $1816)	--	−0.40 (−0.85 to 0.05)	--	SMT Dominant‡	SMT Dominant‡
**Disability reduction (SMD)**	4	650	$1915 ($1093 to $3225)	0%	$1746 ($1668 to $1999)	73%	0.05 (−0.11 to 0.21)	51%	--	--
CLBP	--	200	$2501 ($388 to $4551)	--	$1965 ($1788 to $2127)	--	0.19 (−0.09 to 0.48)	--	$13,163	$10,343
CNP I	--	127	$524 (-$2164 to $2966)	--	$1647 ($1548 to $1756)	--	0.21 (−0.12 to 0.56)	--	$2,494	$7,845
SLBP	--	161	$433 (-$3192 to $4191)	--	$1769 ($317 to $3358)	--	0.08 (−0.26 to 0.40)	--	$5,410	$22,112
SNP	--	162	$2235 ($771 to $3458)	--	$1394 ($573 to $1816)	--	−0.28 (−0.59 to 0.01)	--	SMT Dominant‡	SMT Dominant‡

† Bias-corrected bootstrap confidence intervals; ‡ Dominant = lower mean costs and better mean outcomes; ICER = Incremental cost-effectiveness ratio; QALY = quality adjusted life year; CLBP = chronic low back pain trial in adults; CNP I = first chronic neck pain trial in adults; SLBP = chronic low back pain trial in older adults (seniors); SMD = Standardized Mean Difference; SNP = chronic neck pain trial in older adults (seniors)

**Fig. 3:**
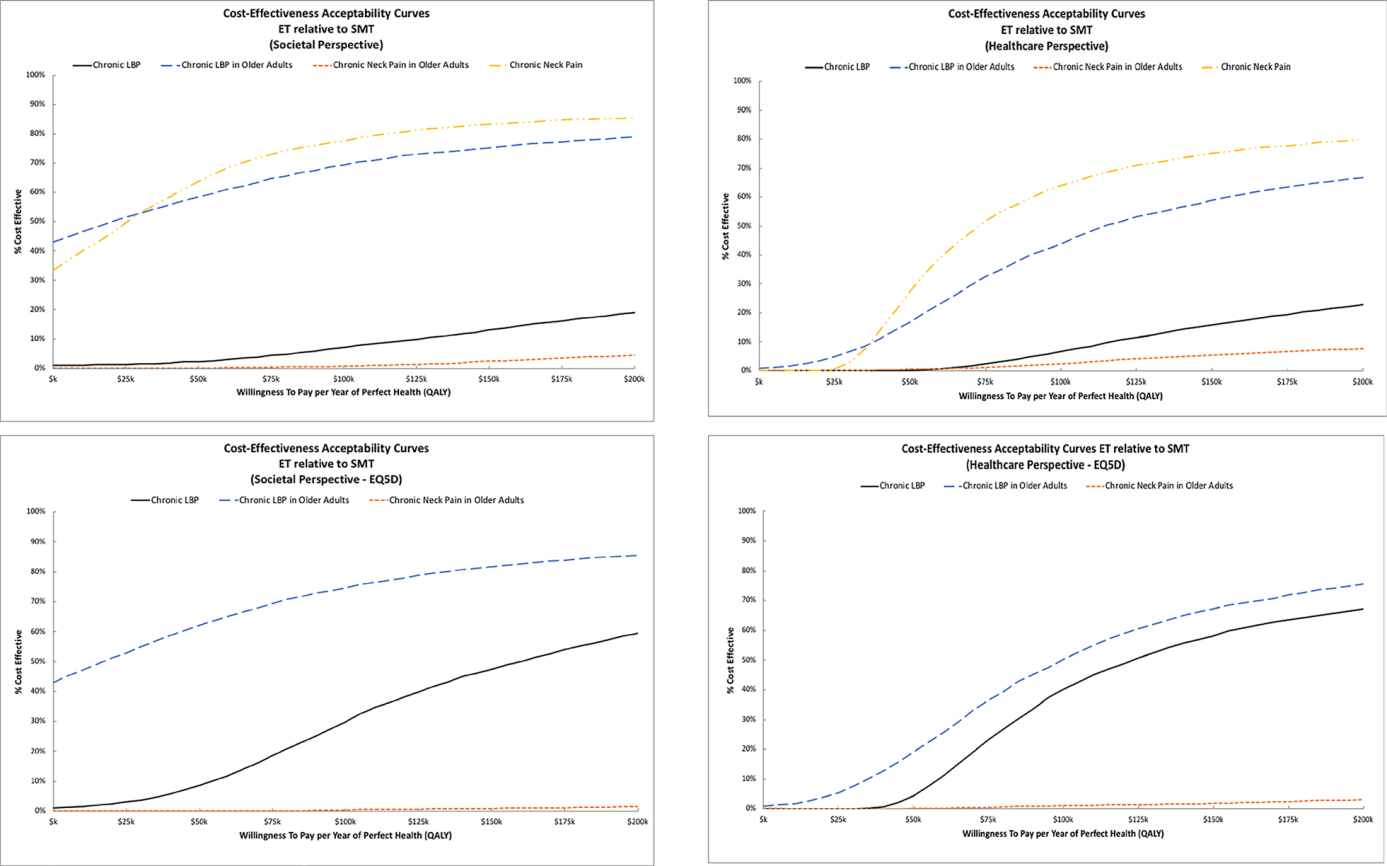
Cost-effectiveness acceptability curves for Exercise Therapy relative to Spinal Manipulative Therapy. The top row displays findings from the primary analysis. The bottom row displays findings from the sensitivity analysis using EQ5D for QALYs. The societal perspective is shown in the left column and the healthcare perspective is shown in the right column

ICER estimates and cost-effectiveness acceptability curves varied by trial. For adults with chronic back pain, ET resulted in higher costs and lower QALYs than SMT across perspectives. The sensitivity analyses using EQ5D QALYs favored ET over SMT, resulting in ICERs of $156k/QALY for the societal perspective and $123k/QALY for the healthcare perspective. Findings for older adults with chronic back pain were more consistent, with ET consistently resulting in higher costs and QALYs with ICERs below $30k/QALY from the societal perspective and below $120k/QALY from the healthcare perspective. For adults with chronic neck pain, ET had higher costs and greater QALYs with ICERs near $22k/QALY from the societal perspective and $69k/QALY from the healthcare perspective. Equation 5D QALY estimates were not available from this trial. For older adults with chronic neck pain, ET resulted in higher costs and lower QALYs from both perspectives. Figure 3 displays the cost-effectiveness acceptability curves for ET relative to SMT. The probability of cost-effectiveness for ET relative to SMT was low (< 20%) across WTP thresholds and perspectives for adults with chronic back pain and older adults with chronic neck pain. The probability of cost-effectiveness for ET in adults with chronic back pain increased in the sensitivity analyses using EQ5D QALYs. For older adults with chronic back pain, the probability of cost-effectiveness for ET was near 70% for WTP thresholds of $100k/QALY and higher from the societal perspective and near 60% for thresholds of $150k/QALY and higher from the healthcare perspective. The sensitivity analysis with EQ5D QALYs had little impact on results from this trial. For adults with chronic neck pain, the probability of cost-effectiveness was near 80% for WTP thresholds of $100k/QALY and higher from the societal perspective and thresholds of $150k/QALY and higher from the healthcare perspective.

### SMT + ET vs. ET

Two trials with 365 participants included data for this comparison (Table 6) [20, 21]. One trial included adolescents with chronic low back pain and the other included adults with chronic neck pain. Complete cost and clinical outcome data was available for 273 (88%) participants. The trial of adolescents with back pain did not capture lost productivity thus societal costs for this trial are limited to formal and informal healthcare sector costs (Table 2). Cost-effectiveness outcomes were not pooled due to heterogeneity between studies in estimated ICERs and cost-effectiveness acceptability curves (e.g. ICERs for societal perspective using EQ5D near $35k/QALY for adolescents with chronic back pain and $150k/QALY for adults with chronic neck pain). QALYs in the adolescent trial were available only from the EQ5D. For adolescents with chronic back pain, adding SMT to ET resulted in mean pain reduction of 0.75 (95% CI 0.30 to 1.23) and a standardized mean disability reduction of 0.29 (95% CI 0.02 to 0.59). Differences in other clinical outcomes were small and not statistically significant in the individual trials or pooled analyses. All differences in clinical outcomes favored adding SMT to ET except pain reduction for adults with chronic neck pain (mean difference − 0.09; 95% CI −0.57 to 0.39). Societal and healthcare costs were higher for adding SMT to ET in both trials. For adolescents with chronic back pain, societal costs were $354 higher (95% CI $210 to $483), and healthcare costs were $280 higher (95% CI $167 to $389). For adults with chronic neck pain, societal costs were $460 higher (95% CI -$2884 to $3421) and healthcare costs were $187 higher (-$435 to $543).

**Table 6:** Cost-effectiveness results for adding spinal manipulative therapy to exercise therapy

	# trials	# subjects	Δ Societal Costs (95% CI)†	I^2^	Δ Healthcare Costs (95% CI) †	I^2^	Δ Outcome (95%CI) †	I^2^	ICER (Societal)	ICER (Healthcare)
**QALYs (SF6D)**										
CNP II	--	180	$460 (-$2884 to $3421)	--	$187 (-$435 to $543)	--	0.006 (−0.019 to 0.031)	--	$76,642/QALY	$31,091/QALY
**QALYs (EQ5D)**	2	365	$354 (-$842 to $1249)	0%	$277 ($22 to $382)	6%	0.003 (−0.024 to 0.033)	0%	--	--
ALBP	--	185	$354 ($210 to $483)	--	$280 ($167 to $389)	--	0.010 (−0.009 to 0.028)	--	$35,356/QALY	$27,981/QALY
CNP II	--	180	$460 (-$2884 to $3421)	--	$187 (-$435 to $543)	--	0.003 (−0.024 to 0.033)	--	$153,282/QALY	$62,180/QALY
**Pain reduction**	2	365	$354 (-$842 to $1249)	0%	$277 ($22 to $382)	6%	0.33 (−0.02 to 0.67)	0%	--	--
ALBP	--	185	$354 ($210 to $483)	--	$280 ($167 to $389)	--	0.75 (0.30 to 1.23)	--	$472	$373
CNP II	--	180	$460 (-$2884 to $3421)	--	$187 (-$435 to $543)	--	−0.09 (−0.57 to 0.39)	--	ET Dominant‡	ET Dominant‡
**Disability reduction (SMD)**	2	365	$354 (-$842 to $1249)	0%	$277 ($22 to $382)	6%	0.23 (0.03 to 0.46)	1%	--	--
ALBP	--	185	$354 ($210 to $483)	--	$280 ($167 to $389)	--	0.29 (0.02 to 0.59)	--	$1,219	$965
CNP II	--	180	$460 (-$2884 to $3421)	--	$187 (-$435 to $543)	--	0.16 (−0.11 to 0.45)	--	$2,874	$1,166

† Bias-corrected bootstrap confidence intervals; ‡ Dominant = lower mean costs and better mean outcomes; ICER = Incremental cost-effectiveness ratio; QALY = quality adjusted life year; ALBP = chronic low back pain trial in adolescents; CNP II = second chronic neck pain trial in adults; SMD = Standardized Mean Difference

**Fig. 4:**
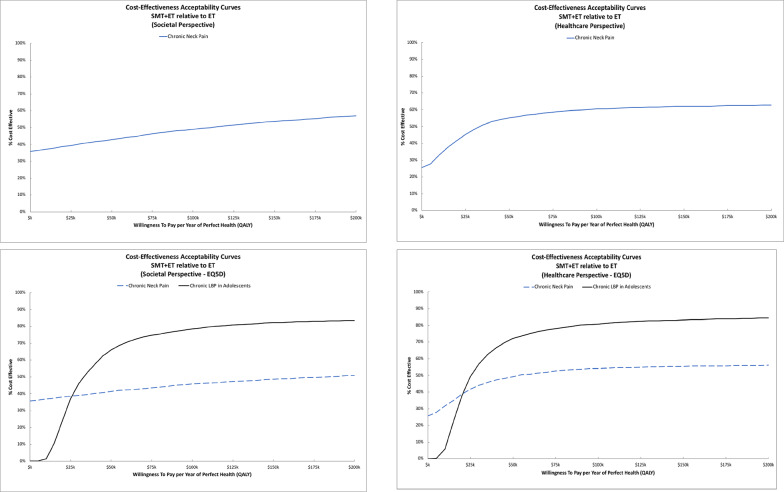
Cost-effectiveness acceptability curves for adding Spinal Manipulative Therapy to Exercise Therapy. The top row displays findings from the primary analysis. The bottom row displays findings from the sensitivity analysis using EQ5D for QALYs. The societal perspective is shown in the left column and the healthcare perspective is shown in the right column

ICER estimates and cost-effectiveness acceptability curves varied between trials. For adults with chronic neck pain, adding SMT to ET resulted in higher costs and QALYs with ICERs of $77k/QALY from the societal perspective and $31k/QALY from the healthcare perspective. QALY gains were lower in the sensitivity analysis using EQ5D, resulting in larger ICERs of $153k/QALY for the societal perspective and $62k/QALY from the healthcare perspective. For adolescents with chronic back pain, adding SMT to ET also resulted in higher costs and QALYs with ICERs of $35k/QALY from the societal perspective and $28k/QALY from the healthcare perspective. Figure 4 displays the cost-effectiveness acceptability curves for adding SMT to ET. For adults with chronic neck pain, the probability of cost-effectiveness is near 50% across perspectives for WTP thresholds above $100k/QALY. The sensitivity analyses using EQ5D QALYs had minimal impact on these findings. For adolescents with chronic back pain, the probability adding SMT to ET is cost-effective is above 70% for WTP thresholds above $50k/QALY across perspectives.

### ET vs. HEA

Two trials with 380 participants included data for this comparison (Table 7) [19, 20]. One trial included adults with chronic neck pain and the other included adults with chronic low back pain. Complete cost and clinical outcome data was available for 282 (74%) participants. Cost-effectiveness outcomes were not pooled due to heterogeneity between studies in estimated ICERs. Differences between treatments in clinical outcomes were small and often not statistically significant. Clinical outcomes favored ET over HEA, except for QALYs measured using the SF6D for adults with chronic low back pain. ET resulted in significantly higher pain reduction relative to HEA in adults with chronic neck pain (mean difference of 0.64; 95% CI 0.15 to 1.16). Pooled differences in pain reduction also significantly favored ET over HEA (mean difference of 0.41; 95% CI of 0.07 to 0.64). Societal and healthcare costs were significantly higher for ET relative to HEA in both studies and the pooled estimates. Mean societal costs ranged from $2583 to $4876 higher and mean healthcare costs were approximately $2200 higher.

**Table 7:** Cost-effectiveness results for exercise therapy relative to home exercise and advice

	# trials	# subjects	Δ Societal Costs (95% CI)†	I^2^	Δ Healthcare Costs (95% CI) †	I^2^	Δ Outcome (95%CI) †	I^2^	ICER (Societal)	ICER (Healthcare)
**QALYs (SF6D)**	2	380	$3704 ($2132 to $5274)	43%	$2271 ($2035 to $2632)	0%	0.001 (−0.018 to 0.018)	21%	--	--
CLBP	--	201	$2583 ($115 to $4808)	--	$2276 ($2134 to $2404)	--	−0.009 (−0.036 to 0.015)	--	HEA Dominant‡	HEA Dominant‡
CNP II	--	179	$4876 ($2821 to $7775)	--	$2185 ($1826 to $2805)	--	0.011 (−0.015 to 0.036)	--	$443,284/QALY	$198,670/QALY
**QALYs (EQ5D)**	2	380	$3704 ($2132 to $5274)	43%	$2271 ($2035 to $2632)	0%	0.013 (−0.007 to 0.032)	0%	--	--
CLBP	--	201	$2583 ($115 to $4808)	--	$2276 ($2134 to $2404)	--	0.005 (−0.023 to 0.32)	--	$515,679/QALY	$455,293/QALY
CNP II	--	179	$4876 ($2821 to $7775)	--	$2185 ($1826 to $2805)	--	0.022 (−0.007 to 0.049)	--	$221,640/QALY	$99,335/QALY
**Pain reduction**	2	380	$3704 ($2132 to $5274)	43%	$2271 ($2035 to $2632)	0%	0.41 (0.07 to 0.73)	32%	--	--
CLBP	--	201	$2583 ($115 to $4808)	--	$2276 ($2134 to $2404)	--	0.23 (−0.23 to 0.64)	--	$11,233	$9,898
CNP II	--	179	$4876 ($2821 to $7775)	--	$2185 ($1826 to $2805)	--	0.64 (0.15 to 1.16)	--	$7,619	$3,414
**Disability reduction (SMD)**	2	380	$3704 ($2132 to $5274)	43%	$2271 ($2035 to $2632)	0%	0.06 (−0.14 to 0.25)	0%	--	--
CLBP	--	201	$2583 ($115 to $4808)	--	$2276 ($2134 to $2404)	--	0.04 (−0.21 to 0.30)	--	$64,585	$56,912
CNP II	--	179	$4876 ($2821 to $7775)	--	$2185 ($1826 to $2805)	--	0.08 (−0.22 to 0.37)	--	$60,952	$27,318

† Bias-corrected bootstrap confidence intervals; ‡ Dominant = lower mean costs and better mean outcomes; ICER = Incremental cost-effectiveness ratio; QALY = quality adjusted life year; CLBP = chronic low back pain trial in adults; CNP II = second chronic neck pain trial in adults; SMD = Standardized Mean Difference

**Fig. 5:**
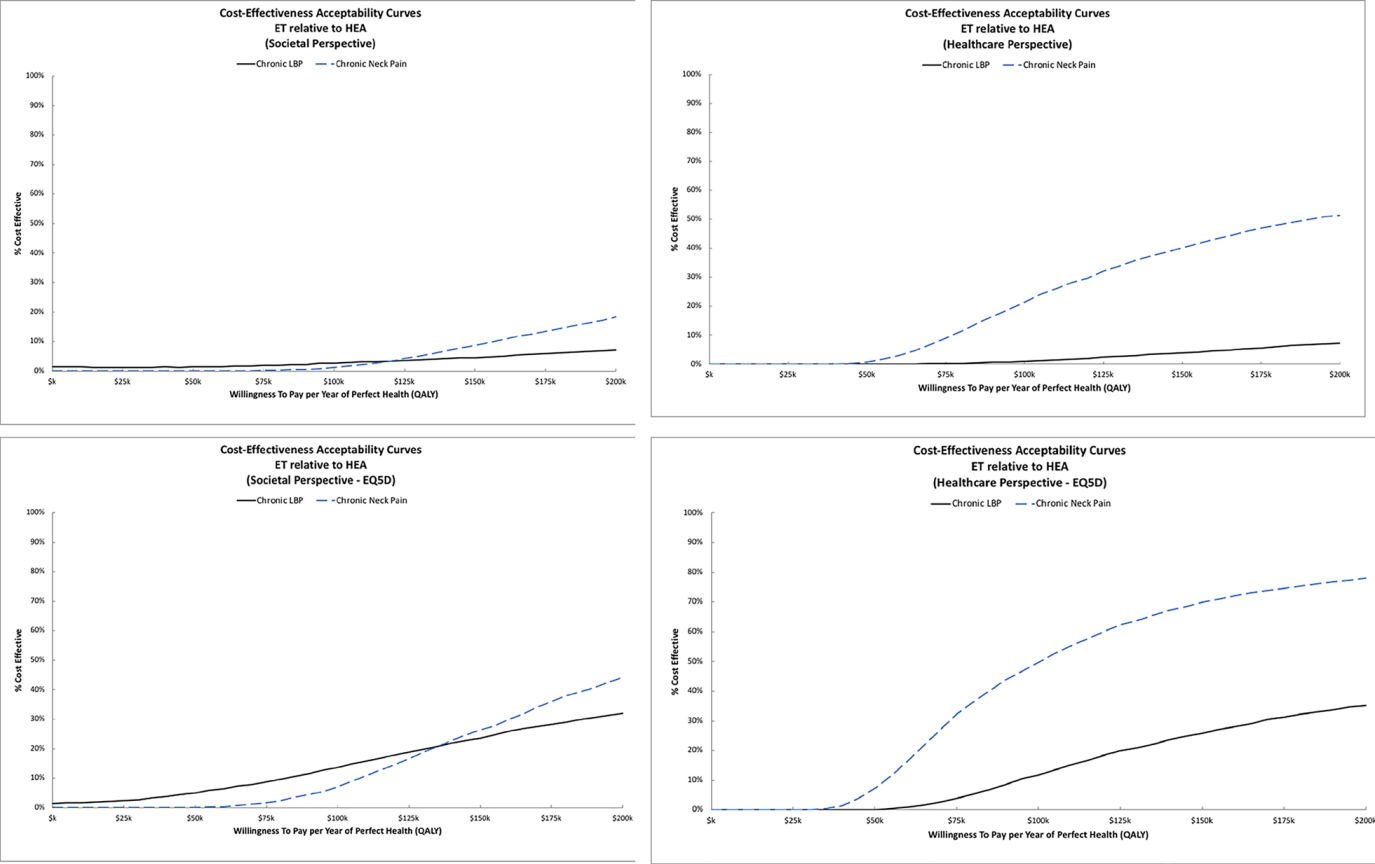
Cost-effectiveness acceptability curves for Exercise Therapy relative to Home Exercise and Advice. The top row displays findings from the primary analysis. The bottom row displays findings from the sensitivity analysis using EQ5D for QALYs. The societal perspective is shown in the left column and the healthcare perspective is shown in the right column

ICER estimates varied between trials. For adults with chronic back pain, ET cost more and was less effective for the primary analysis using SF6D QALYs. In the sensitivity analysis using the EQ5D for QALYs, ICERs were above $400k/QALY. Figure 5 shows cost-effectiveness acceptability curves for ET relative to HEA. The probability of cost-effectiveness was very low (< 10%) across perspectives and WTP thresholds and remained below 30% across WTP thresholds in the sensitivity analyses using EQ5D QALYs. For chronic neck pain, ET relative to HEA resulted in an ICER above $400k/QALY from the societal perspective and just under $200k/QALY from the healthcare perspective. In the sensitivity analysis using EQ5D for QALYs, the ICERs reduced to $222k/QALY from the societal perspective and to $99k/QALY from the healthcare perspective. The probability of cost-effectiveness was low (< 20%) across WTP thresholds from the societal perspective. From the healthcare perspective, the probability of cost-effectiveness increased from approximately 20% at a WTP threshold of $100k/QALY to 50% at a threshold of $200k/QALY. From the societal perspective, in the sensitivity analyses using EQ5D QALYs, the probability of cost-effectiveness was under 45% across WTP thresholds. From the healthcare perspective, the probability of cost-effectiveness increased to 50% at a WTP threshold of $100k/QALY and near 80% at a threshold of $200k/QALY.

### ET + HEA vs. HEA

Two trials with 321 participants included data for this comparison (Table 8) [22, 23]. One trial included older adults with chronic low back pain, and the other included older adults with chronic neck pain. Complete cost and clinical outcome data was available for 289 (90%) participants. Cost-effectiveness outcomes were not pooled due to heterogeneity between studies in estimated ICERs and cost-effectiveness acceptability curves. Differences between treatments in clinical outcomes were small and not statistically significant within the individual trials or pooled analyses. For older adults with chronic back pain, clinical outcomes favored adding ET to HEA for QALYs and disability, but not pain reduction. For older adults with chronic neck pain, the only clinical outcome favoring adding ET to HEA was pain intensity. Societal and healthcare costs were consistently higher when adding ET to HEA. Differences in societal costs were not statistically significant and ranged from $1382 higher for LBP to $1692 higher for neck pain. Differences in healthcare costs were significantly higher when adding ET to HEA and ranged from $2791 higher for LBP (95% CI of $1821 to $4448) to $2117 higher for neck pain (95% CI $1924 to $2363).

**Table 8:** Cost-effectiveness results for adding exercise therapy (ET) to home exercise and advice (HEA)

	# trials	# subjects	Δ Societal Costs (95% CI)†	I^2^	Δ Healthcare Costs (95% CI) †	I^2^	Δ Outcome (95%CI) †	I^2^	ICER (Societal)	ICER (Healthcare)
**QALYs (SF6D)**	2	321	$1606 (-$388 to $3488)	0%	$2157 ($1768 to $2545)	6%	0.003 (−0.015 to 0.020)	0%	--	--
SLBP	--	160	$1382 (-$2490 to $4763)	--	$2791 ($1821 to $4448)	--	0.008 (−0.019 to 0.034)	--	$172,714/QALY	$348,818/QALY
SNP	--	161	$1694 (-$659 to $3710)	--	$2117 ($1924 to $2363)	--	−0.0004 (−0.023 to 0.022)	--	HEA Dominant‡	HEA Dominant‡
**QALYs (EQ5D)**	2	321	$1606 (-$388 to $3488)	0%	$2157 ($1768 to $2545)	6%	−0.001 (−0.015 to 0.014)	0%	--	--
SLBP	--	160	$1382 (-$2490 to $4763)	--	$2791 ($1821 to $4448)	--	0.005 (−0.027 to 0.024)	--	$276,343/QALY	$558,108/QALY
SNP	--	161	$1694 (-$659 to $3710)	--	$2117 ($1924 to $2363)	--	−0.007 (−0.014 to 0.14)	--	HEA Dominant‡	HEA Dominant‡
**Pain reduction**	2	321	$1606 (-$388 to $3488)	0%	$2157 ($1768 to $2545)	6%	0.07 (−0.27 to 0.40)	0%	--	--
SLBP	--	160	$1382 (-$2490 to $4763)	--	$2791 ($1821 to $4448)	--	−0.03 (−0.52 to 0.47)	--	HEA Dominant‡	HEA Dominant‡
SNP	--	161	$1694 (-$659 to $3710)	--	$2117 ($1924 to $2363)	--	0.16 (−0.29 to 0.61)	--	$10,588	$13,231
**Disability reduction (SMD)**	2	321	$1606 (-$388 to $3488)	0%	$2157 ($1768 to $2545)	6%	0.07 (−0.14 to 0.29)	1%	--	--
SLBP	--	160	$1382 (-$2490 to $4763)	--	$2791 ($1821 to $4448)	--	0.18 (−0.09 to 0.49)	--	$7,676	$15,503
SNP	--	161	$1694 (-$659 to $3710)	--	$2117 ($1924 to $2363)	--	−0.04 (−0.35 to 0.27)	--	HEA Dominant‡	HEA Dominant‡

† Bias-corrected bootstrap confidence intervals; ‡ Dominant = lower mean costs and better mean outcomes; ICER = Incremental cost-effectiveness ratio; QALY = quality adjusted life year; SLBP = chronic low back pain trial in older adults (seniors); SMD = Standardized Mean Difference; SNP = chronic neck pain trial in older adults (seniors)

**Fig. 6:**
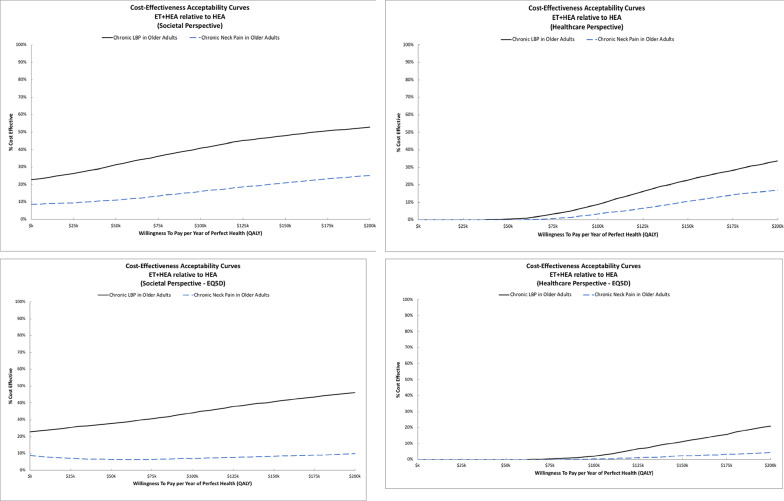
Cost-effectiveness acceptability curves for adding Exercise Therapy to Home Exercise and Advice. The top row displays findings from the primary analysis. The bottom row displays findings from the sensitivity analysis using EQ5D for QALYs. The societal perspective is shown in the left column and the healthcare perspective is shown in the right column

ICER estimates and cost-effectiveness acceptability curves varied between trials. For older adults with chronic neck pain, adding ET to HEA cost more and was less effective for improving QALYs from both the societal and healthcare perspective. Sensitivity analyses with EQ5D QALYs produced similar results. Figure 6 shows cost-effectiveness acceptability curves for adding ET to HEA. The probability of cost-effectiveness was low (< 20%) across perspectives and WTP thresholds. For older adults with chronic back pain, adding ET to HEA resulted in an ICERs of $173k/QALY from the societal perspective and above $300k/QALY from the healthcare perspective. In the sensitivity analysis using EQ5D for QALYs, the ICERs increased to $276k/QALY from the societal perspective and above $500k/QALY from the healthcare perspective. For the societal perspective, the probability of cost-effectiveness was near 40% at a WTP threshold of $100k/QALY and increased to 50% at the $170k/QALY threshold. The sensitivity analysis using EQ5D QALYs reduced these probabilities by a small amount to near 35% at a WTP threshold of $100k/QALY that increased to around 45% at a threshold of $200k/QALY. The probability of cost-effectiveness was below 30% across WTP thresholds for the healthcare perspective.

## Discussion

### Summary of findings

Our objective was to estimate the cost-effectiveness of SMT, ET, and HEA for spinal pain in the U.S. with an IPDMA approach using data from eight RCTs with similar methods, outcomes, comparisons, and settings. Despite these similarities and low heterogeneity for pooled effectiveness outcomes, estimates of cost-effectiveness and their uncertainty varied considerably across trials preventing meta-analysis of cost-effectiveness outcomes. We planned a limited number of sub-group analyses to explore potential sources of heterogeneity, but did not perform them due to the limited number of participants in subgroups for each planned comparison. Instead, we reported cost-effectiveness results by trial. Table 9 provides a high-level summary of findings considering ICER results in relationship to potential willingness-to-pay thresholds for a QALY, the probability of cost-effectiveness across the range of willingness-to-pay thresholds, and the consistency of findings across perspectives and outcomes.

**Table 9.**
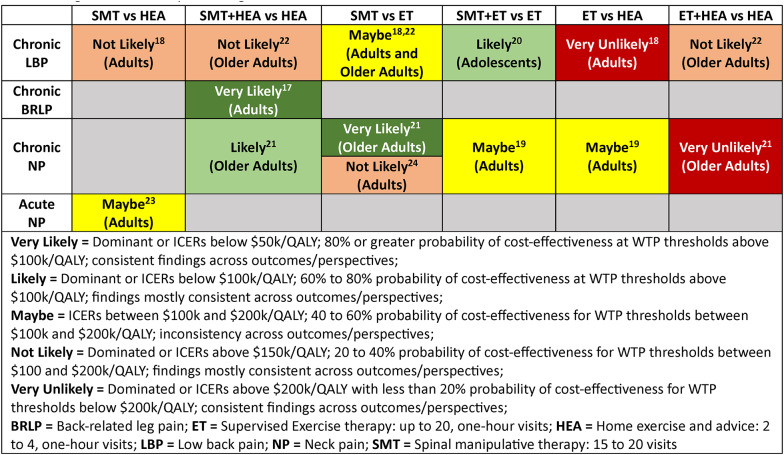
High-level summary of findings

### SMT vs. HEA

Acute neck pain: SMT may be cost-effective depending on outcome and perspective. SMT had higher costs and better outcomes on average, but differences in outcomes were small. QALY estimates differed considerably between the SF6D and EQ5D with ICERs from $19k to $44k/QALY using the SF6D and $131k to $305k/QALY using EQ5D. ICERs were more favorable for the healthcare perspective.

Chronic back pain: SMT resulted in higher costs and worse health outcomes across all outcomes and perspectives and is unlikely to be cost-effective.

### SMT + HEA vs. HEA

Chronic neck pain in older adults: Adding SMT to HEA is also likely to be cost-effective as it resulted in better health outcomes and lower societal costs. Healthcare costs were higher, but ICERs were below $100k/QALY.

Chronic back pain in older adults: Adding SMT to HEA is not likely cost-effective. It led to fewer QALYs, small differences in pain and disability reduction, and higher costs.

Back-related leg pain: Adding SMT to HEA resulted in lower costs and better health outcomes across all outcomes and perspectives and is very likely cost-effective.

### ET vs. SMT

Chronic neck pain: SMT is not likely cost-effective relative to ET as ET consistently resulted in better outcomes and higher costs, with ICERs below $70k/QALY.

Chronic neck pain in older adults: SMT is very likely cost-effective as it consistently resulted in lower costs and better outcomes with high probabilities (> 90%) of cost-effectiveness across a wide range of WTP thresholds.

Chronic back pain: SMT may be cost-effective as costs were consistently higher for ET and QALY estimates differed considerably between the SF6D and EQ5D, resulting in ICERs that ranged from domination for SMT (SF6D) to as low as $122k/QALY for ET (EQ5D).

Chronic back pain in older adults: SMT may be cost-effective as findings were inconsistent across outcomes. ET resulted in higher QALYs and costs with ICERs below $29k/QALY from the societal and $118k/QALY from the healthcare perspective, but findings for other outcomes were inconsistent.

#### SMT + ET vs. ET

Chronic neck pain: Adding SMT to ET may be cost-effective with ICERs below $77k/QALY (SF6D) and $154k/QALY (EQ5D), but findings for other outcomes were inconsistent.

Chronic back pain in adolescents: Adding SMT to ET results in higher costs and better health outcomes and is likely cost-effective.

### ET vs. HEA

Chronic neck pain: ET may be cost-effective depending on outcome and perspective. The most favorable results were for QALYs and pain from the healthcare perspective. QALY estimates differed considerably between the SF6D and EQ5D for chronic neck pain with healthcare perspective ICERs near $200k/QALY (SF6D) and $100k/QALY (EQ5D).

Chronic back pain: ET is very unlikely cost-effective as there were consistently higher costs (>$2000) with either worse outcomes or small improvements with ICERs above $450k/QALY.

#### ET + HEA vs. HEA

Chronic neck pain in older adults: Adding ET to HEA is very unlikely to be cost-effective as costs were consistently higher with worse health outcomes.

Chronic back pain in older adults: Adding ET to HEA is not likely cost-effective as costs were consistently higher with high ICERs.

#### Strengths

The use of randomized trial data lowers the risk of selection bias in differences in clinical outcomes, healthcare use, and costs. Multiple high-quality systematic reviews, including Cochrane reviews, have assessed the included trials as fair to high quality [13, 65–69]. The amount of missing data was low with 83% of participants providing complete cost and clinical outcome data. The similarity between trials in treatment comparisons, protocols, settings, and data collection measures and methods is a major strength. We assessed cost-effectiveness from both the healthcare and societal perspective. We also included costs for reduced work both in and outside of the home which is not common for economic evaluations in the spine pain field. Inclusion of multiple QALY measures and condition-specific measures such as pain intensity and disability is also a strength as it allows assessment of consistency in findings across outcomes.

#### Limitations

There are also important limitations to consider when assessing our findings. Randomized clinical trials are often designed to detect important differences in disease-specific clinical outcomes that are most likely to be impacted by the treatments assessed (e.g., pain severity, disability). Important measures for assessing cost-effectiveness include general health outcomes like changes in QALYs, healthcare use, and missed work. These measures were collected alongside disease-specific measures, but the trials were not powered to detect important differences in cost-effectiveness outcomes. Participants self-reported their use of healthcare and medications along with number of missed workdays. We did not have access to administrative data for healthcare use or costs. While access to administrative data would have reduced potential measurement error for these variables, it is not without limitations due to the high variability in coverage and re-imbursement policies for healthcare procedures across insurance products in the U.S. Costs for reduced productivity due to spinal pain included missed work in and outside of the home, but costs due to reduced productivity while still at work (i.e., presenteeism) were not included. This is an important limitation as costs due to reduced productivity while at work consistently account for a large proportion of total costs in spinal pain burden of illness studies [70, 71]. Finally, all studies were conducted in the U.S. with resources valued using U.S. prices and findings are not likely generalizable to populations or healthcare systems in other countries.

### Implications for clinical practice and research

We report incremental cost-effectiveness ratios for three commonly used conservative interventions for managing neck or back pain. Cost-effectiveness of these interventions often varied by population and perspective. Prior cost-effectiveness studies of SMT, ET, HEA, or their combinations have been conducted in European settings where healthcare costs are much lower than the U.S [72]. Overall, these studies generally found SMT to be cost-effective [27–29, 31, 33, 34, 36, 37], while the cost-effectiveness of ET [27, 29, 31, 34, 36] and HEA [28, 30, 33] varied by trial, program format, and population.

One trial compared manual therapy (SMT) (up to six, 45-minute visits) to a home exercise and advice intervention delivered by general practitioners for primarily chronic neck or back pain [37]. Manual therapy had lower healthcare costs, lower societal costs, and higher QALY gains compared to home exercise and advice.

Four trials compared manual therapy (SMT) and exercise interventions for neck pain. A trial by Korthals de Bos primarily included participants with acute or sub-acute neck pain and compared manual therapy (up to six, 45-minute sessions) to physiotherapy (up to twelve, 30 min sessions; primarily exercise) and general practitioner care [27]. Manual treatment had lower healthcare and societal costs and better clinical outcomes compared to physiotherapy or general practitioner care for neck pain. A trial by Bosmans et al. included participants with subacute neck pain and compared manual therapy (up to six, 30–45 min sessions) to a behavioral graded exercise approach (up to eighteen, 30-minute sessions) [29]. Manual therapy resulted in significantly worse pain and disability outcomes, but differences in QALYs, healthcare, and societal costs were small and favored manual therapy. A trial by Van Dongen et al. compared manual therapy (up to six, 30–60 min sessions) to physiotherapy (up to nine, 30 min sessions) for neck pain lasting between 2 and 52 weeks [36]. Manual therapy resulted in lower healthcare and societal costs and better clinical outcomes relative to physiotherapy, but differences in QALYs were small and the probabilities of cost-effectiveness were near 50% across a range of WTP thresholds, indicating that neither approach is cost-effective compared to the other. Finally, a trial by Lewis et al. compared the addition of either manual therapy or pulsed shortwave diathermy to an advice and exercise intervention with up to seven, 20-minute visits allowed for each approach [28]. Adding manual treatment to advice and exercise had the highest probability of being cost-effective compared to the other two approaches across all WTP thresholds from the societal perspective.

Three trials compared manual therapy and exercise interventions for LBP. The UK BEAM trial included participants with acute or chronic LBP and compared an active education intervention in general practice to either a group exercise intervention that included cognitive behavioral principles (nine, 60-minute classes), SMT (up to eight, 20-minute visits), or both exercise and SMT [31]. Compared to the active education intervention, both the SMT and exercise interventions resulted in higher healthcare costs and more QALYs, with ICERs below $19k/QALY. SMT had the highest probability of cost-effectiveness relative to the other treatments at WTP thresholds of $22k/QALY and higher. A trial by Rivero-Arias et al. compared the addition of a physiotherapy intervention including manual therapy and exercise instruction (up six, 30-minute visits) to an active education intervention (one, 60-minute visit) for primarily chronic LBP [33]. The manual therapy and exercise intervention led to higher healthcare costs and QALYs with an ICER less than $7k/QALY. Total societal costs were lower for the manual treatment and exercise intervention. Finally, a trial by Critchley et al. compared a physiotherapy intervention of manual therapy and home exercise instruction (up to twelve, 30-minute visits) to a low back stabilization group exercise program (eight, 90-minute classes) or a pain management program that included education, exercises, and cognitive behavioral therapy approaches (eight, 90-minute classes) for chronic LBP [34]. The pain management program had the lowest healthcare costs and highest QALYs gains. The manual therapy and home exercise intervention resulted in higher healthcare costs and QALYs relative to the exercise intervention with an ICER below $2500/QALY.

Unlike many European countries, in the U.S. no single entity is responsible for healthcare coverage and payment decisions and the use of cost-effectiveness findings directly impacting policy decisions is limited [73]. Decisions regarding cost-effectiveness of interventions in the U.S. are highly context-specific given the fragmented nature of health insurance and healthcare. Consensus does not exist on the threshold values of Dollars per QALY that represent good value for healthcare services. The Panel of Cost-effectiveness in Health and Medicine recommends using a range of thresholds from $50k/QALY to $200k/QALY to assess the value of strategies for promoting health [64]. These ranges coincide with recommendations from the World Health Organization for using two to three times the per capita annual income which would be $140k/QALY to $210k/QALY in the U.S [64]. Compared to home exercise and advice, ICERs for SMT were often below and ICERs for exercise therapy were frequently above recommended WTP thresholds. Importantly, these findings may not easily translate to settings outside the U.S. where costs for individual treatments and resources often differ dramatically [72]. 

While this study adds important information on the cost-effectiveness of SMT, exercise therapy, and home exercise and advice for spinal pain in the U.S., additional studies are needed to assess the cost-effectiveness of these approaches relative to medical care, the most common treatment approach in the US [74, 75], as well as other guideline recommended treatments such as massage, acupuncture, mindfulness-based stress reduction, tai chi, yoga, and cognitive behavioral therapy [76, 77]. Further, trials assessing the cost-effectiveness of common approaches in higher risk populations with more impactful pain and higher resource use (e.g. radiating arm or leg pain, high-impact chronic pain) are needed. Finally, future spinal pain studies should also measure reduced productivity while at work (i.e. presenteeism) as well as missed work, as presenteeism accounts for a substantial portion of the societal burden.

## Conclusions

Meta-analysis was not indicated due to the large variation between studies in cost-effectiveness estimates. Overall based on willingness to pay thresholds of $50-$200k/QALY, there was moderate to high probability that spinal manipulation is cost-effective relative to HEA for neck pain and back-related leg pain, but not for chronic back pain. There was also moderate to high probability spinal manipulation was cost-effective relative to exercise therapy for chronic back pain but findings were mixed for neck pain and more favorable in older adults. Cost-effectiveness findings for exercise therapy were mostly not favorable relative to less intensive home exercise programs as costs were higher, and outcomes were often worse.

## Supplementary Information

Supplementary Material 1

## Data Availability

No datasets were generated or analysed during the current study.

## Abbreviations

CEA: Cost-effectiveness analysis
CEAC: Cost-effectiveness acceptability curve
CPT: Current procedural terminology
ET: Supervised exercise therapy
HCPCS: Healthcare common procedure coding system
HEA: Home exercise and advice
IA: Iowa
ICER: Incremental cost-effectiveness ratio
IPDMA: Individual participant data meta-analysis
MN: Minnesota
NRS: Numerical rating scale
OR: Oregon
PedsQL: Pediatric quality of life inventory
QALY: Quality-adjusted life year
RCT: Randomized clinical trial
SMT: Spinal manipulation therapy
US: United States
WTP: Willingness-to-pay
