# Supplementary material for: Cost-effectiveness of spinal manipulation, exercise, and self-management for spinal pain
Source: Chiropr Man Therap. 2025 Aug 23;33:36. doi: 10.1186/s12998-025-00599-8 (PMC12375277; doi:10.1186/s12998-025-00599-8)

Appendix Materials

Appendix Table 1. Unit cost of healthcare resources		
**Healthcare resource**		**Source**
Study Treatments		
Spinal manipulative therapy visit	$29.99	HCPCS 98940
Supervised rehabilitative exercise visit (60 minutes)	$117.91	HCPCS 97110 (4 units)
Home exercise and advice visit (60 minutes)	$121.24	HCPCS 97110 (3 units) & 97535 (1 unit)
Non-study Treatments		
Chiropractic evaluation	$44.76	HCPCS 99212
Spinal manipulative therapy	$29.99	HCPCS 98940
Heat	$6.60	HCPCS 97010 (1 unit)
Ultrasound	$11.50	HCPCS 97035 (1 unit)
Acupuncture (60 minutes)	$129.38	HCPCS 97810 (1 unit) & 97811 (3 units)
Manual therapy	$23.07	HCPCS 97140 (1 unit)
Physician evaluation	$75.89	HCPCS 99213
Physical therapy evaluation	$46.23	HCPCS 97001
Exercise therapy (30 minutes)	$58.96	HCPCS 97110 (2 units)
Traction	$13.86	HCPCS 97012 (1 unit)
Massage (30 minutes)	$46.14	HCPCS 97124 (2 units)
Spine radiographs	$39.21	HCPCS 72040/72100 Global
Epidural injection	$480.23	HCPCS 62310/62311 Average
MRI	$263.31	HCPCS 72141/72148 Global
Cervical Fusion	$23,698.69	Average Medicare costs for facility, anesthesiologist, and surgeon costs
Lumbar Fusion	$35,569.70	Average Medicare costs for facility, anesthesiologist, and surgeon costs
Medications		Medicare drug profile public use file – average cost/day
Non-narcotic analgesics	$0.81	
NSAID	$1.34	
Muscle Relaxant	$0.85	
Narcotic analgesic	$3.73	
Anti-depressant	$0.39	
HCPCS = Healthcare common procedure coding system		

SMT vs HEA Figures

QALYs (SF6D)

Societal Perspective	Healthcare Perspective

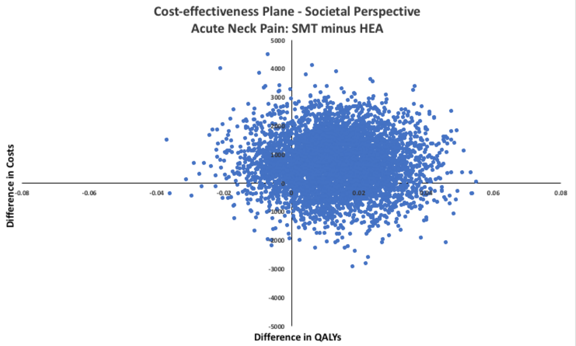

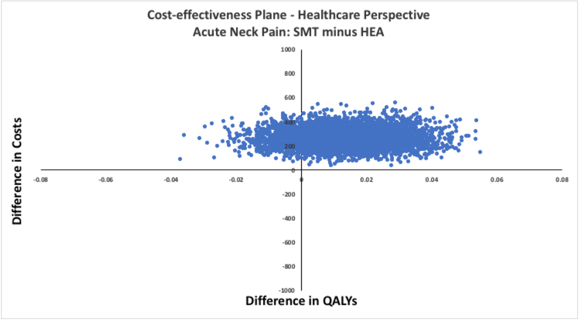

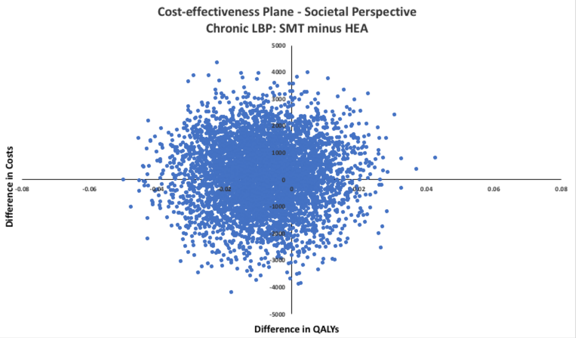

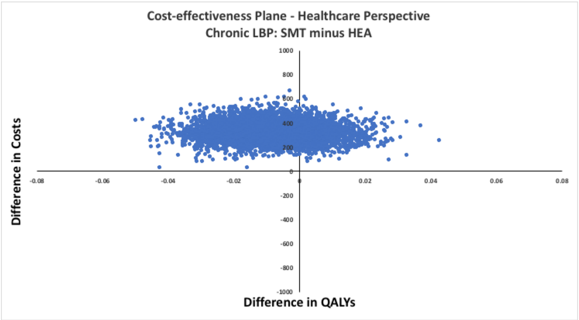

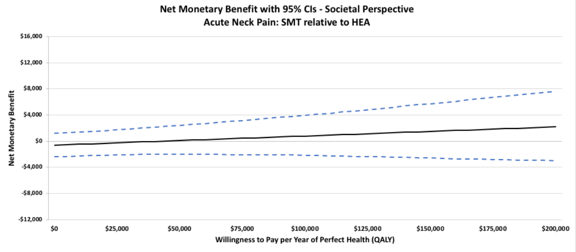

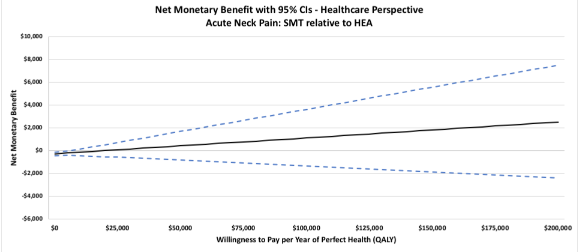

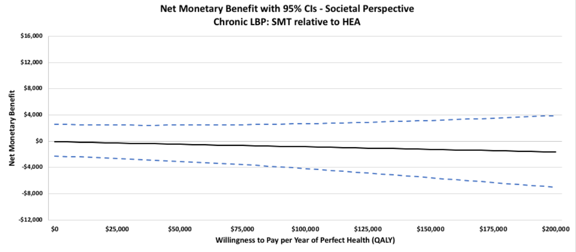

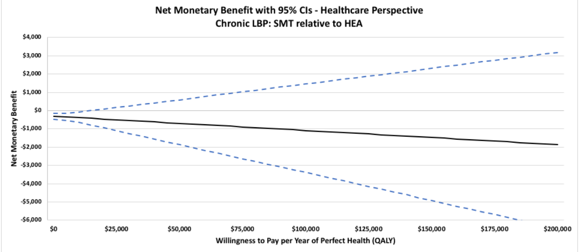

QALYs (EQ5D)

Societal Perspective	Healthcare Perspective

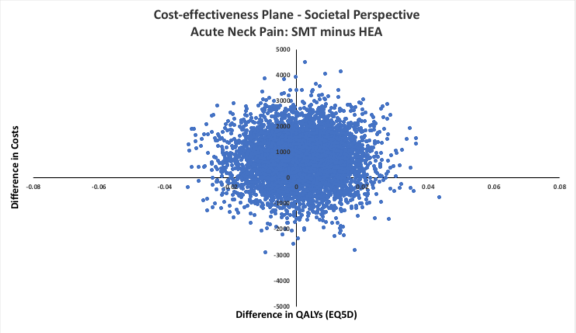

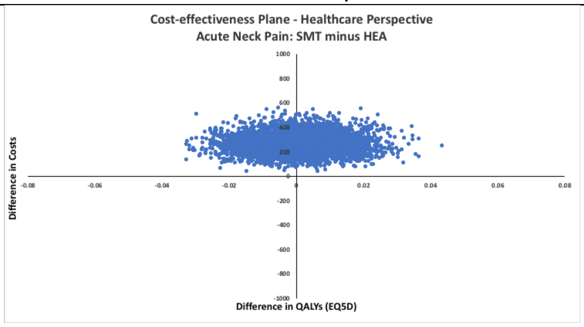

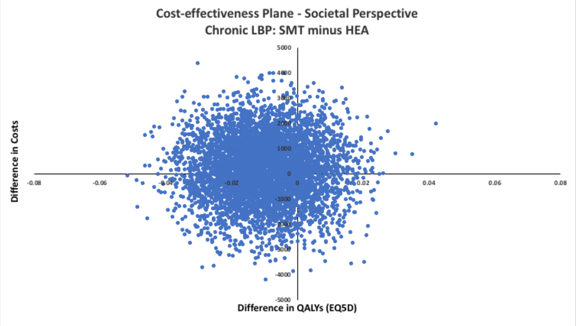

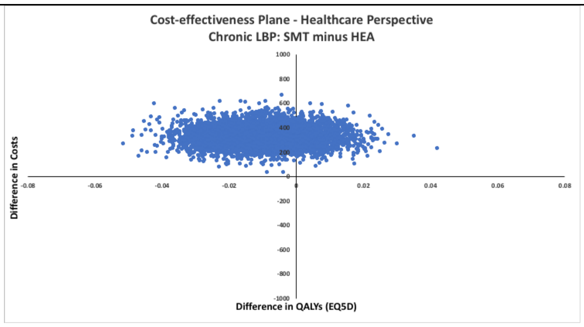

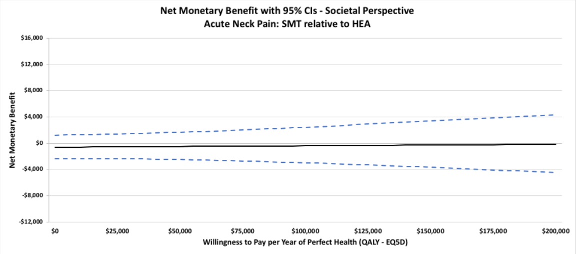

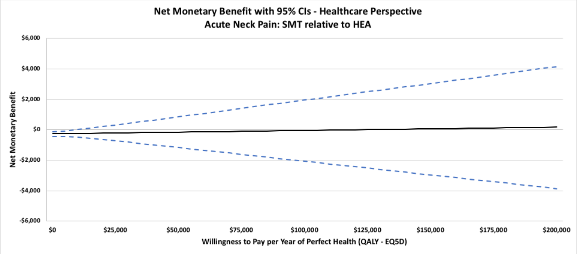

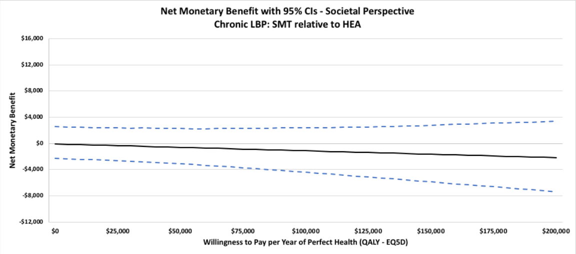

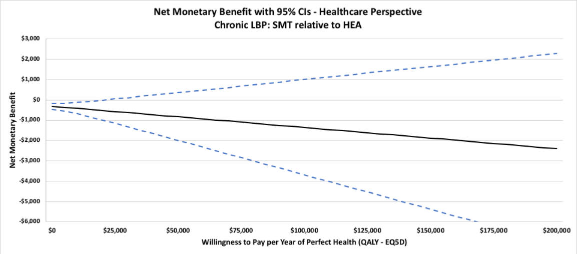

Pain Reduction

<table>
<tr><th>Societal Perspective</th><th>Healthcare Perspective</th></tr>
<tr><td>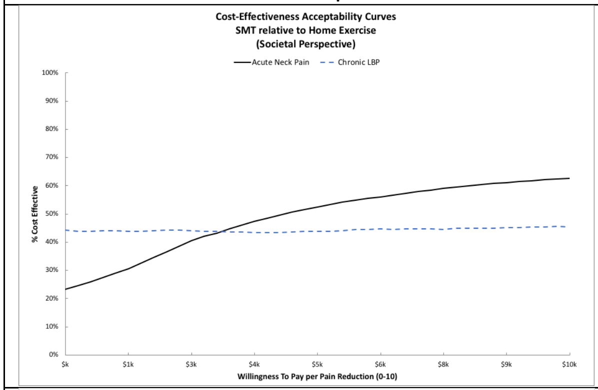
</td><td>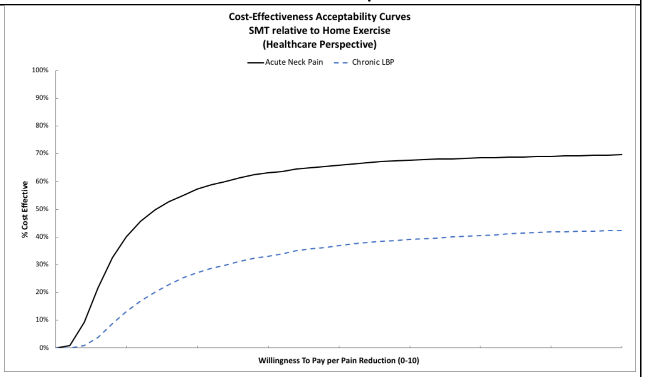
</td></tr>
<tr><td>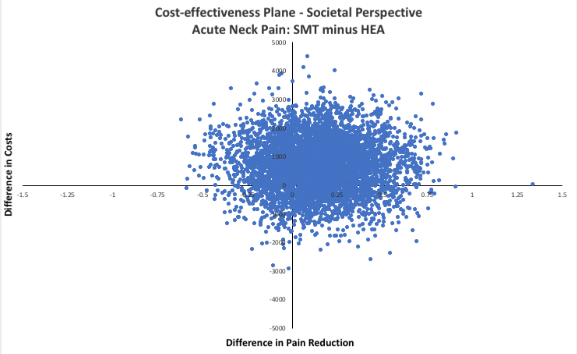
</td><td>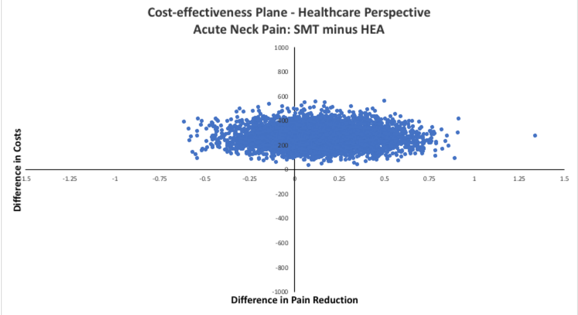
</td></tr>
<tr><td>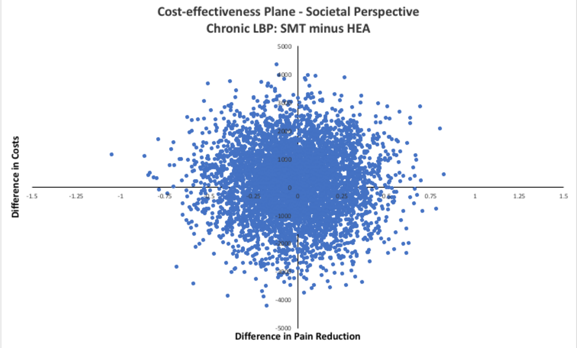
</td><td>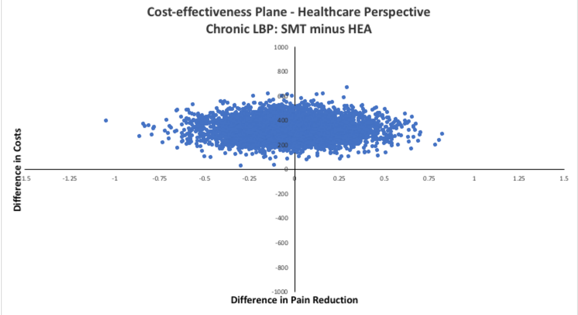
</td></tr>
</table>

Disability Reduction

Societal Perspective	Healthcare Perspective

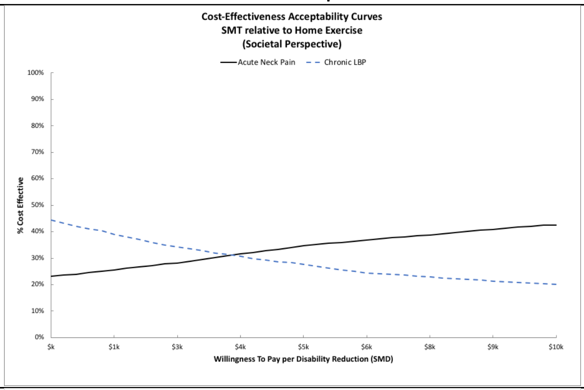

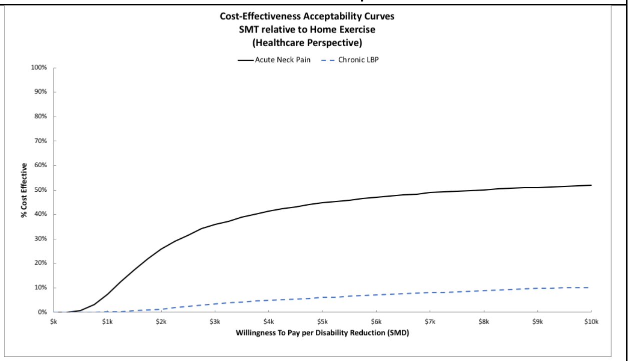

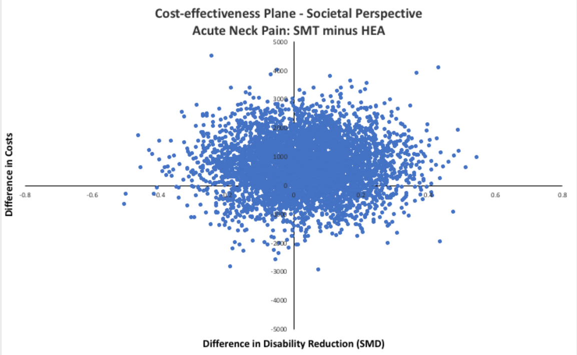

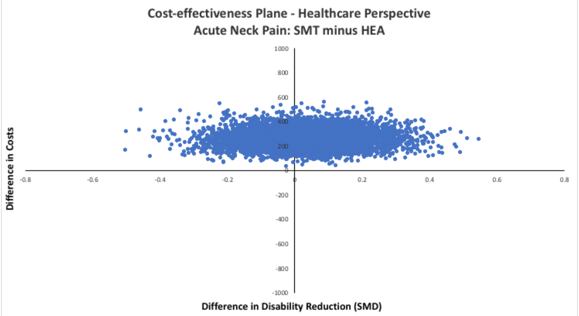

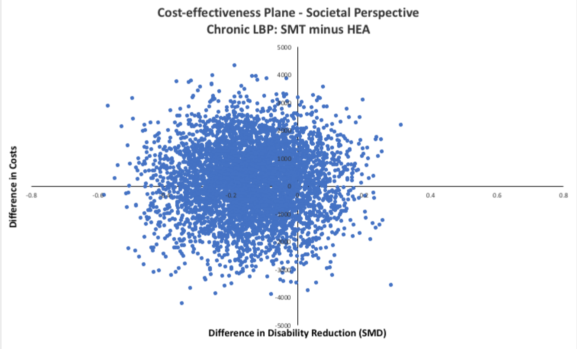

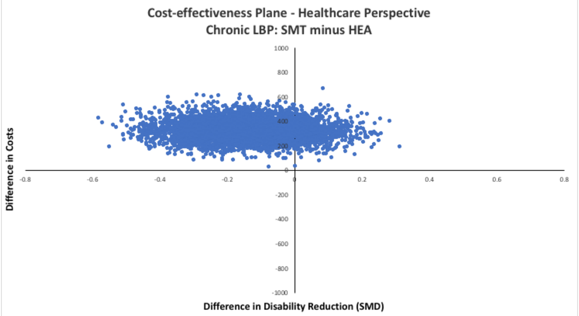

<u>SMT + HEA vs HEA Figures</u>

QALYs (SF6D)

Societal Perspective	Healthcare Perspective
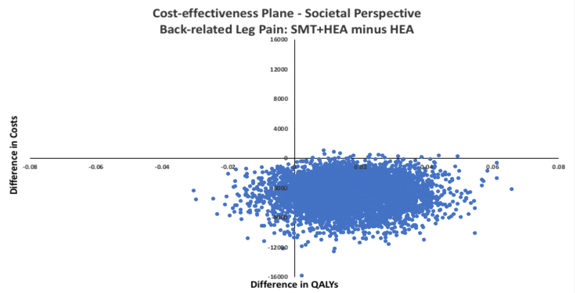 	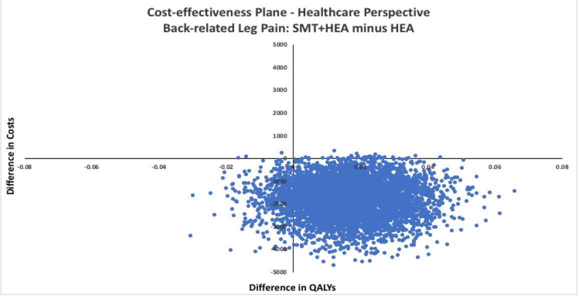
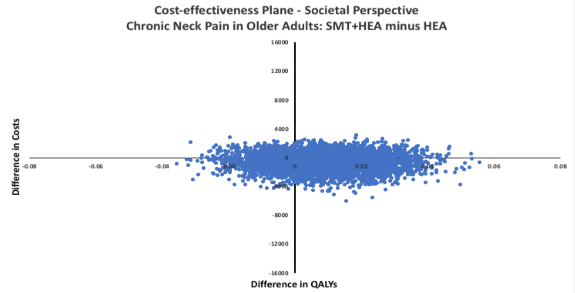 	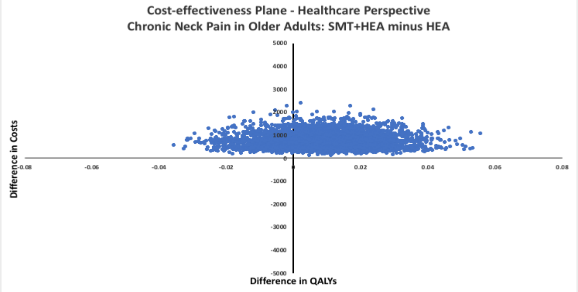
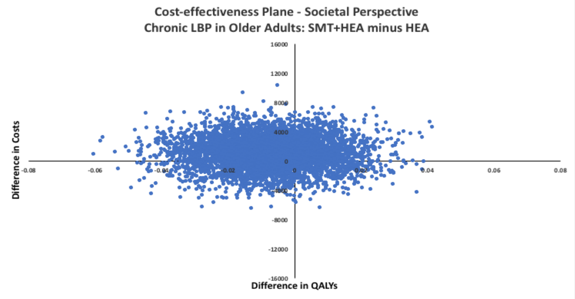 	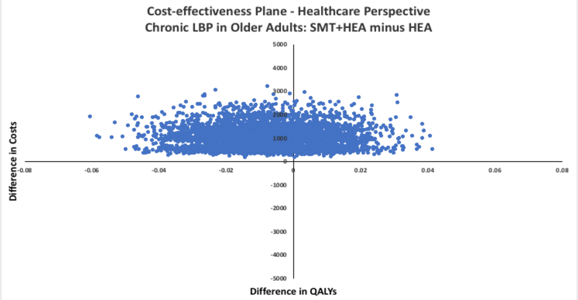

QALYs (SF6D)

Societal Perspective	Healthcare Perspective

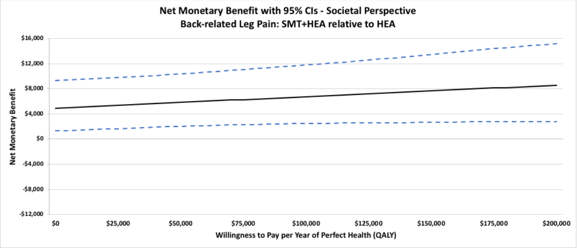

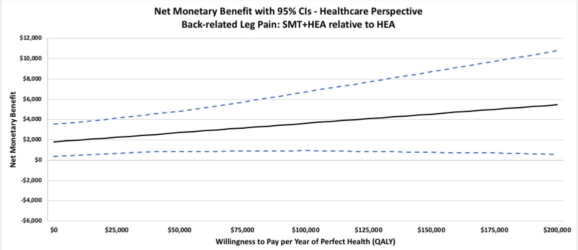

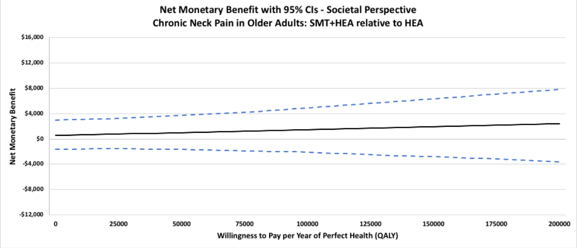

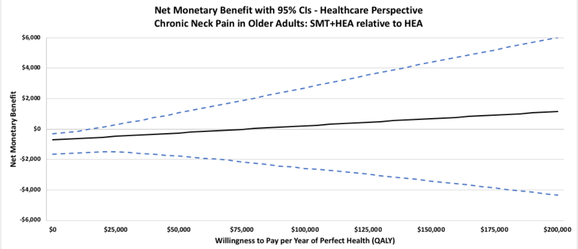

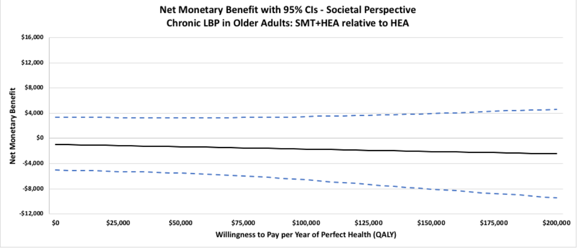

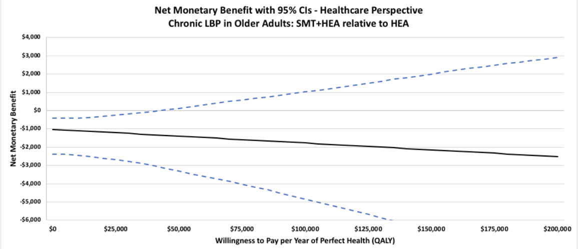

QALYs (EQ5D)

Societal Perspective	Healthcare Perspective

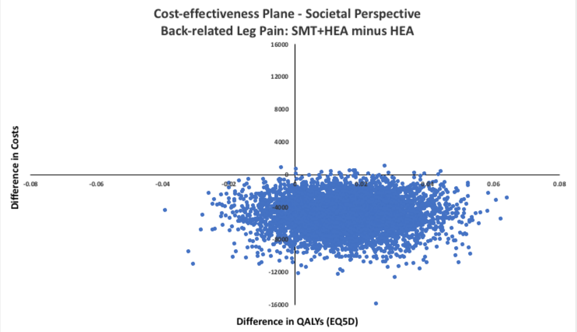

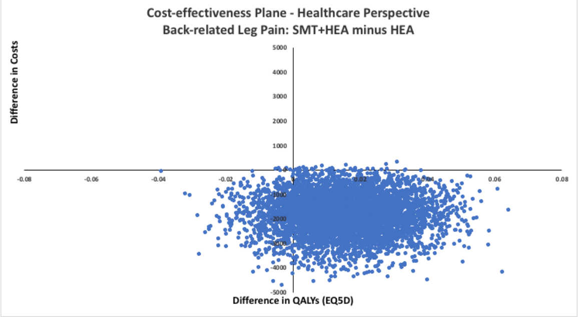

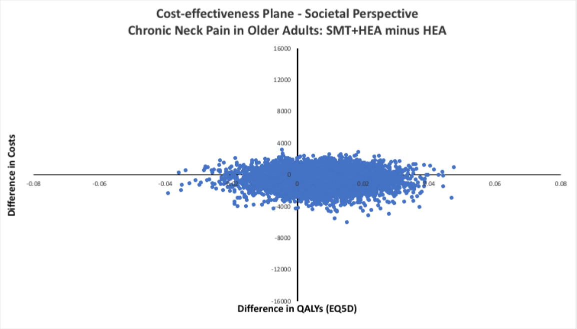

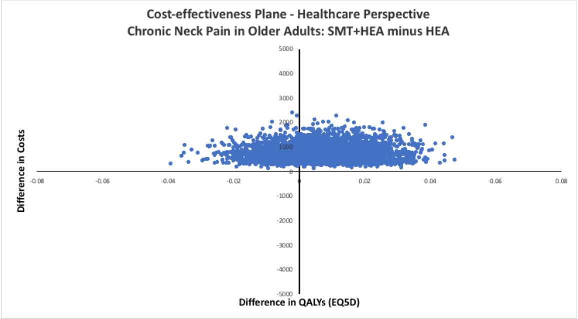

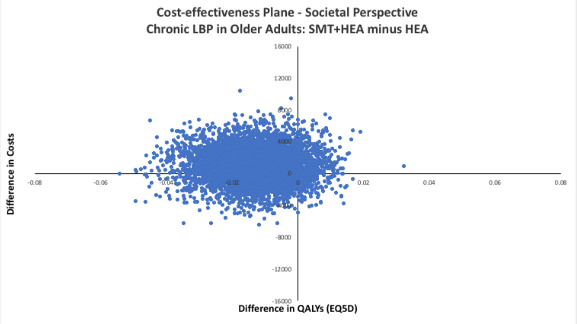

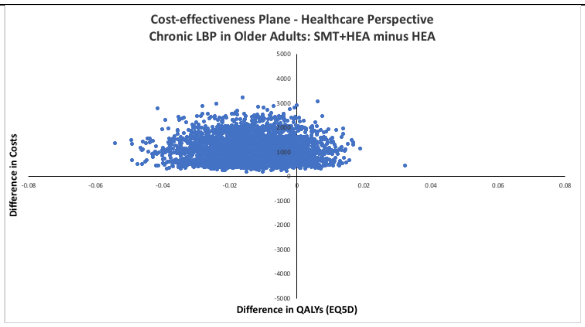

QALYs (EQ5D)

Societal Perspective	Healthcare Perspective

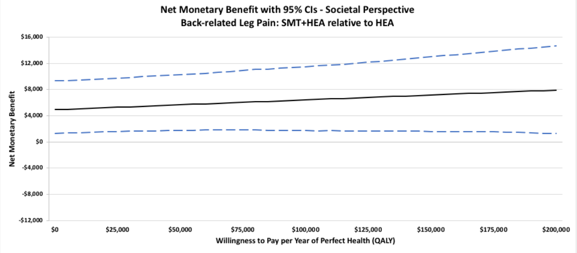

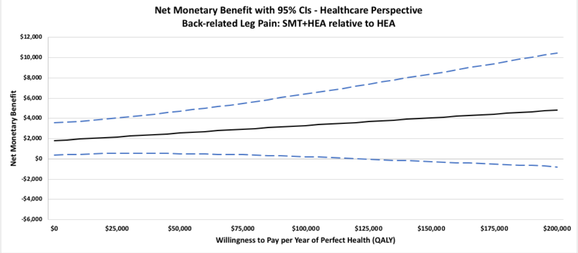

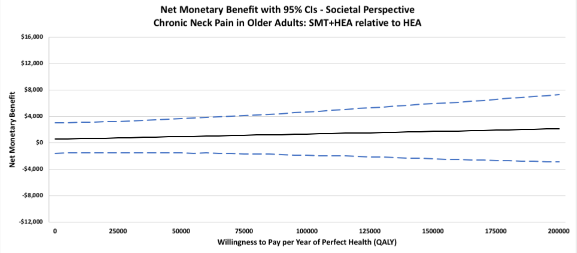

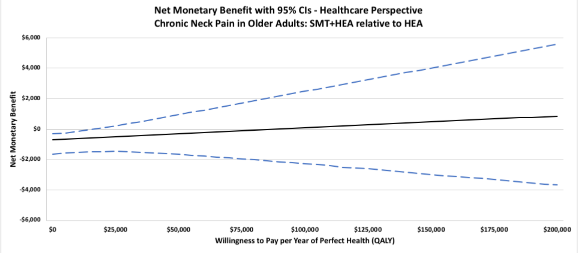

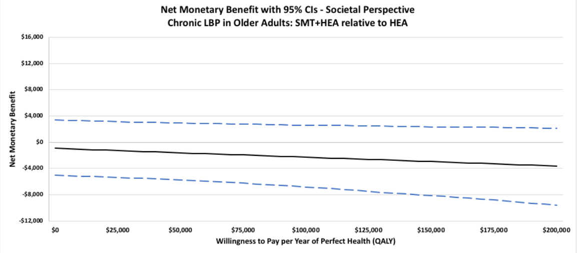

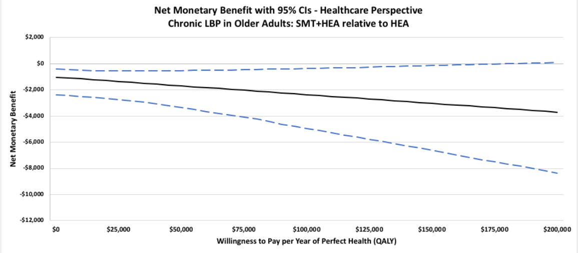

Pain Reduction

Societal Perspective

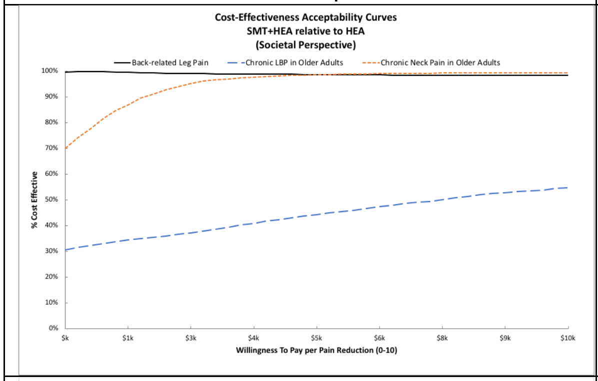

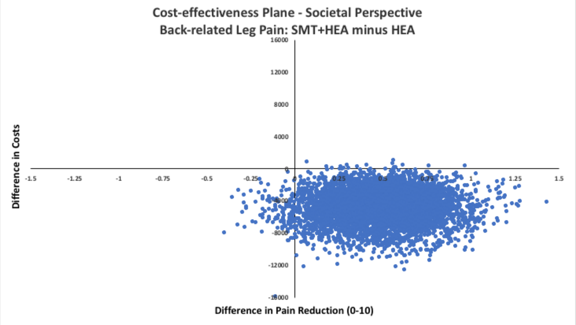

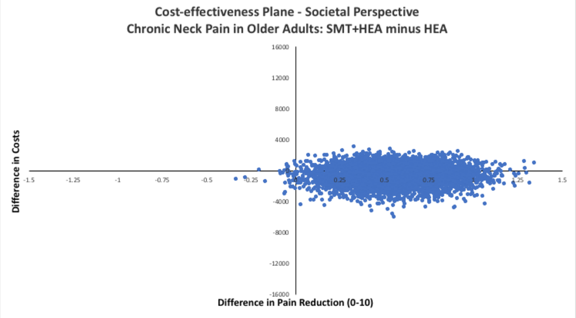

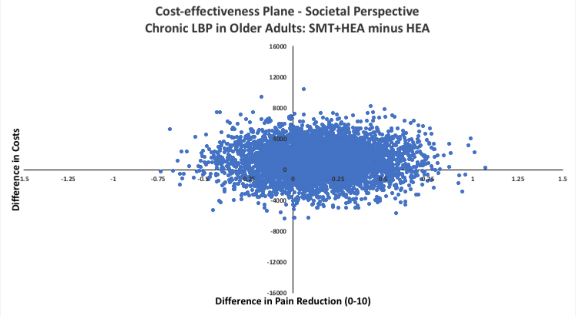

Healthcare Perspective

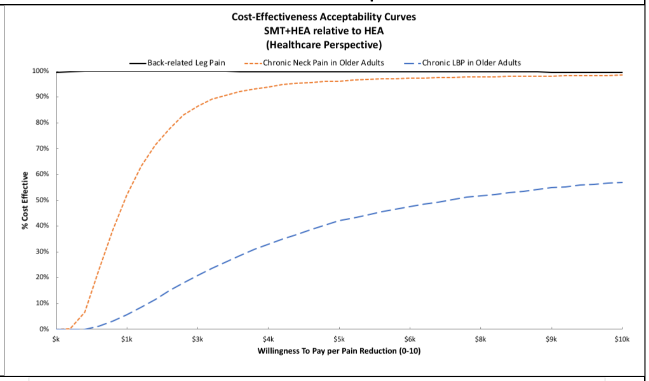

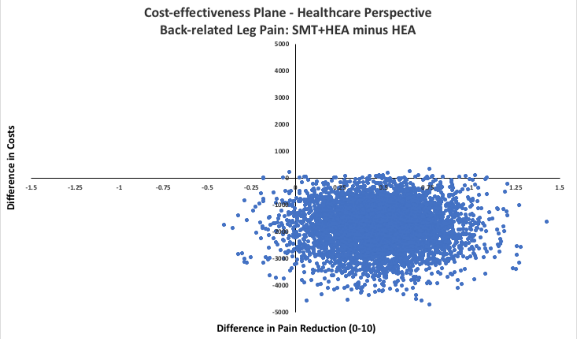

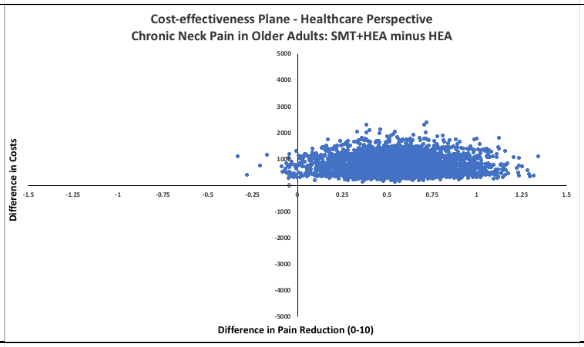

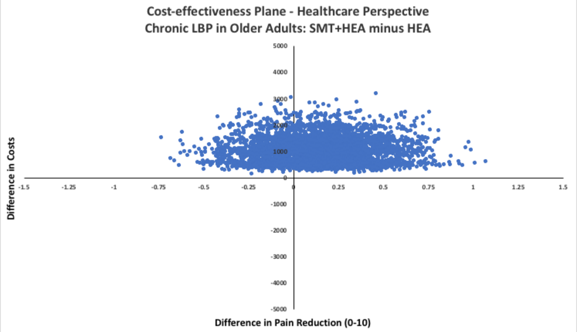

Disability Reduction

Societal Perspective

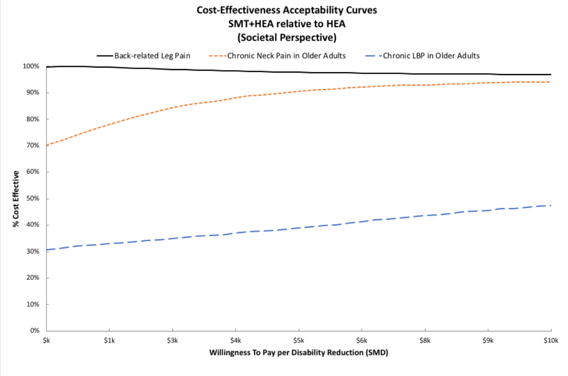

Healthcare Perspective

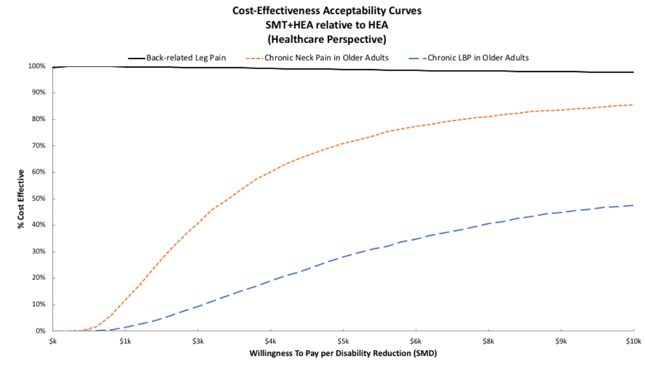

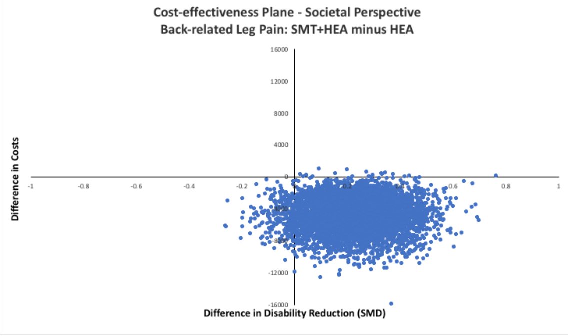

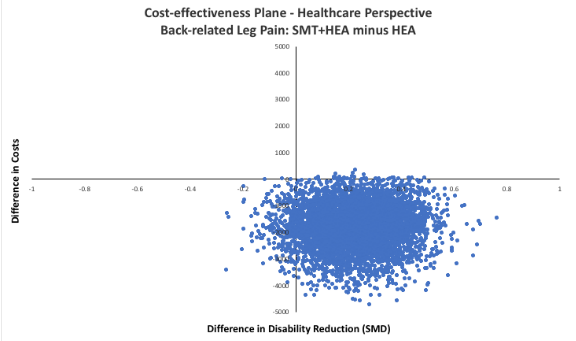

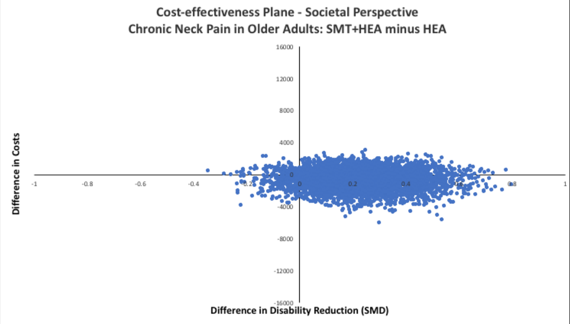

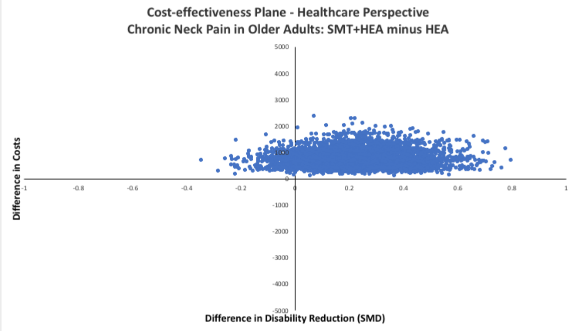

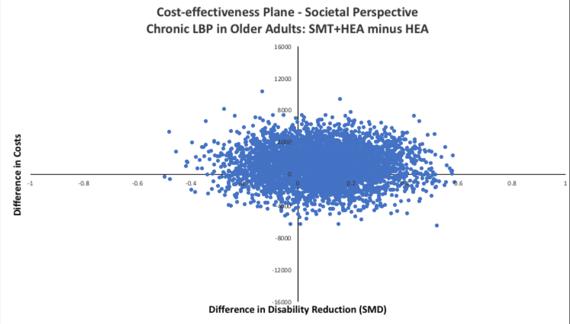

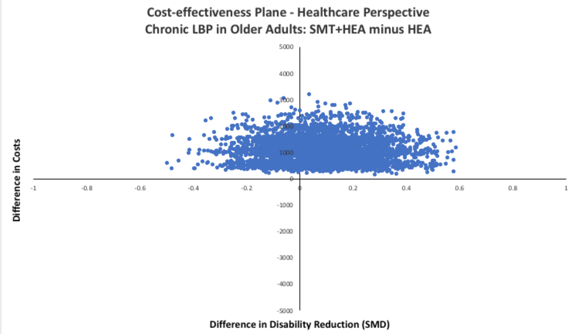

ET vs SMT Figures

QALYs (SF6D)

Societal Perspective	Healthcare Perspective
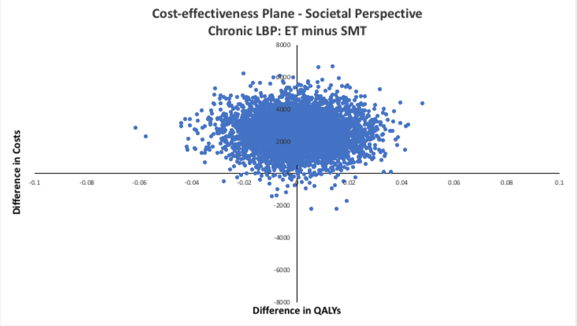	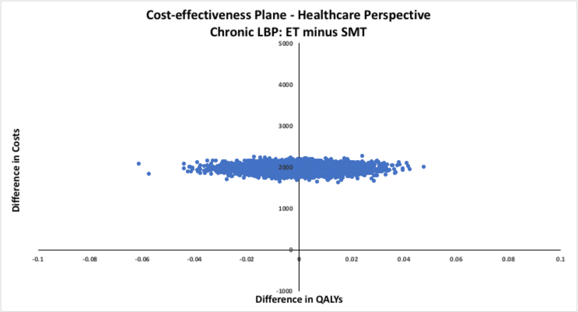
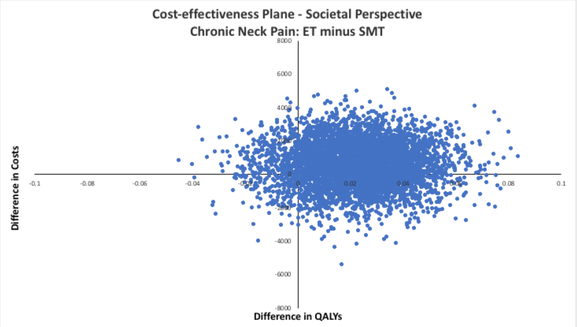	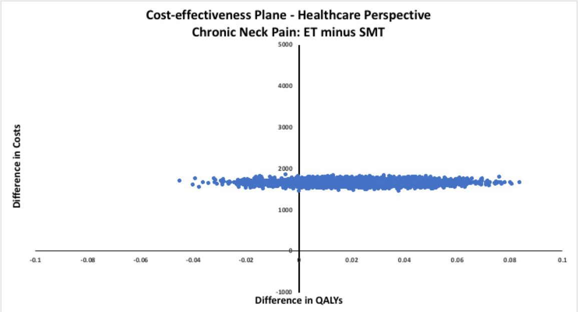
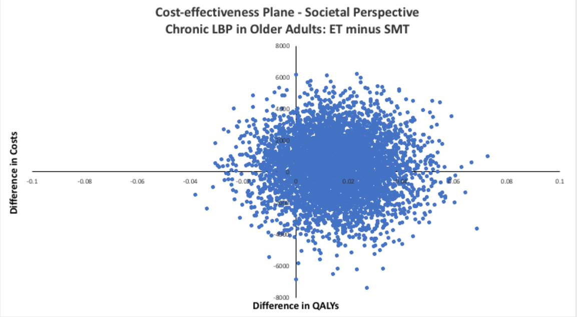	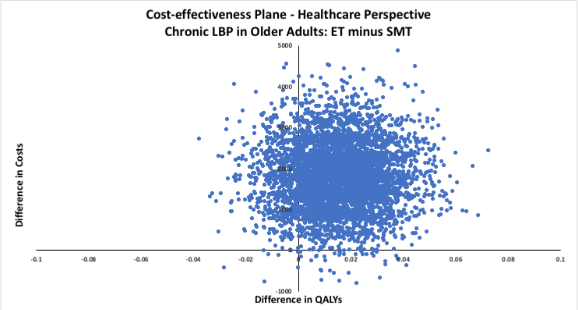
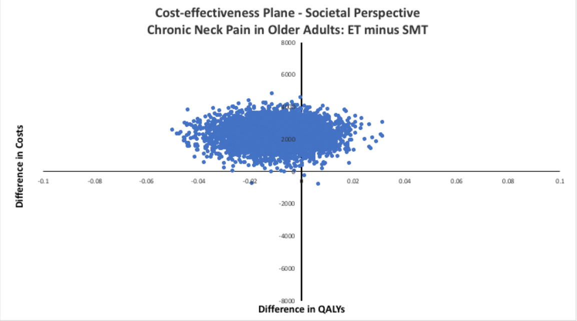	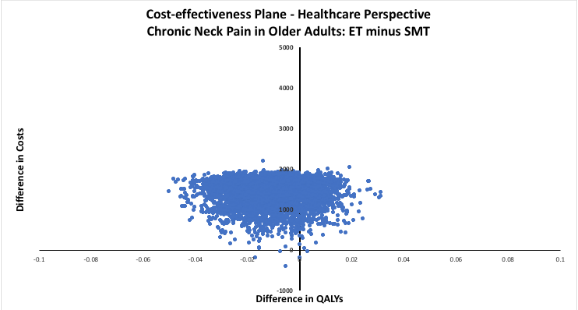

QALYs (SF6D)

Societal Perspective	Healthcare Perspective

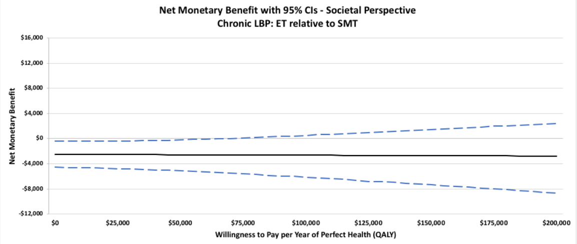

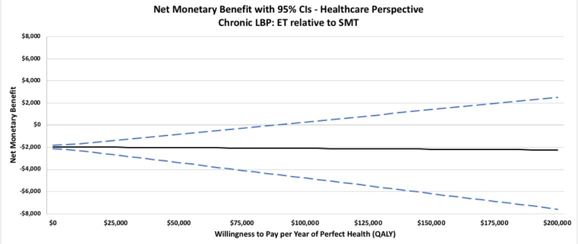

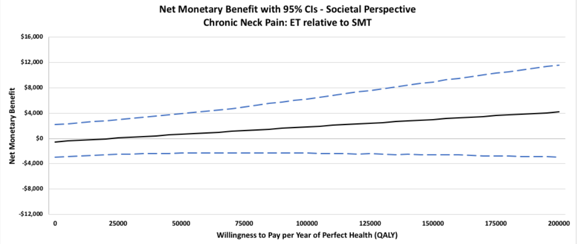

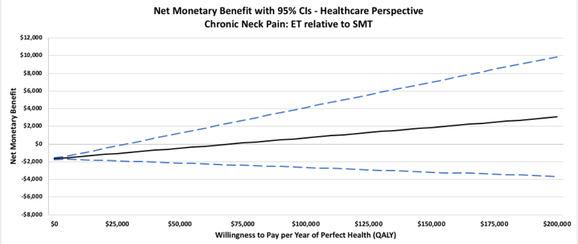

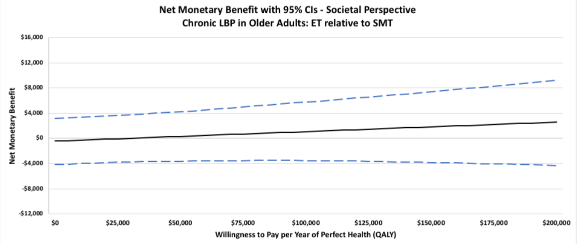

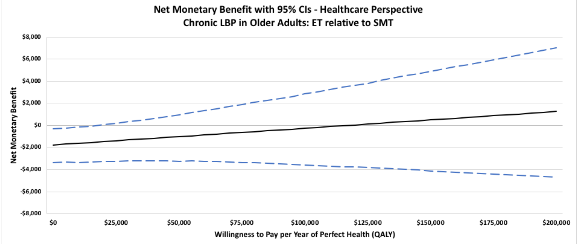

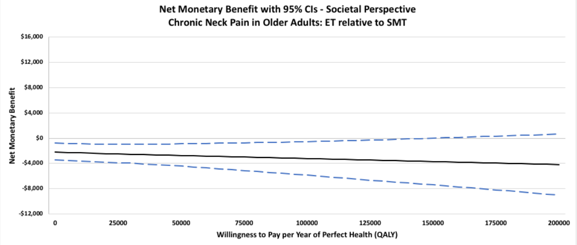

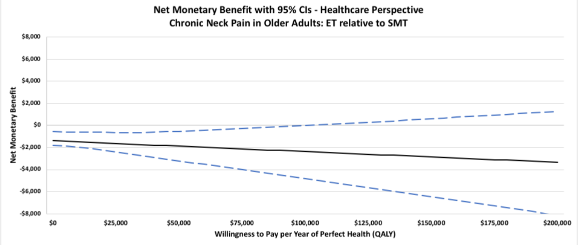

QALYs (EQ5D)

Societal Perspective	Healthcare Perspective
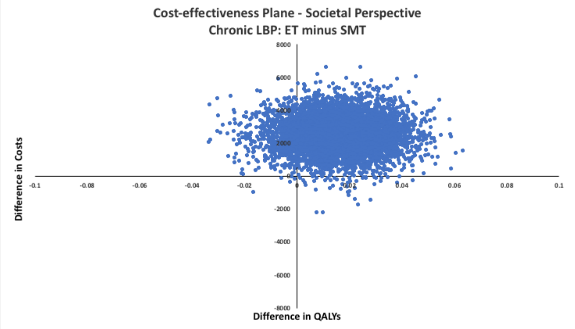	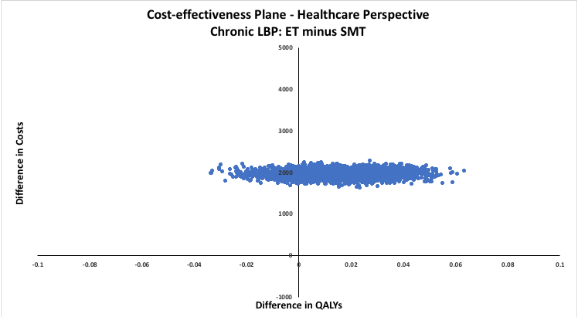
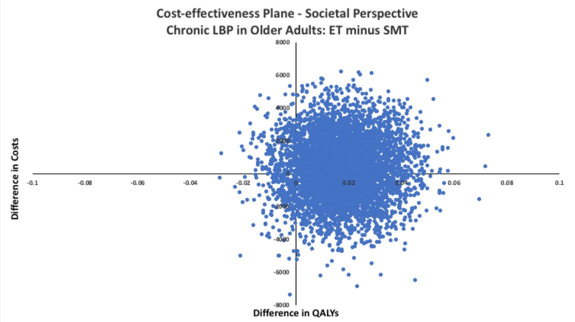	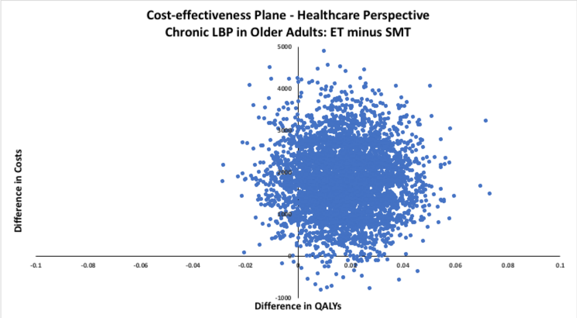
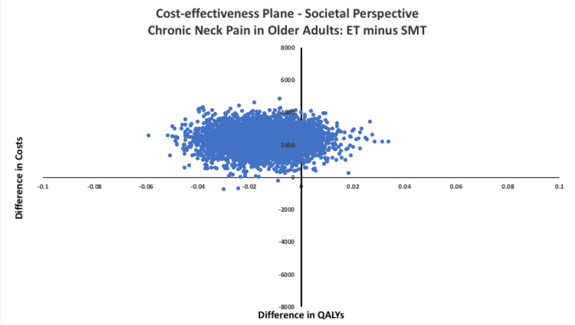	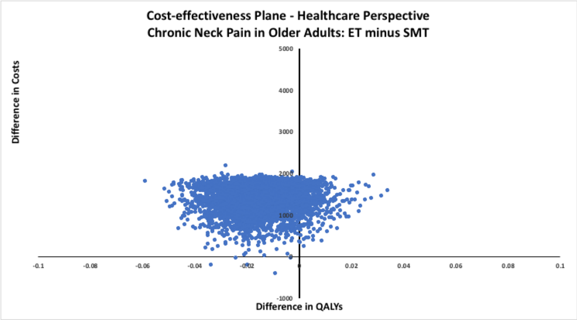

QALYs (EQ5D)

Societal Perspective	Healthcare Perspective

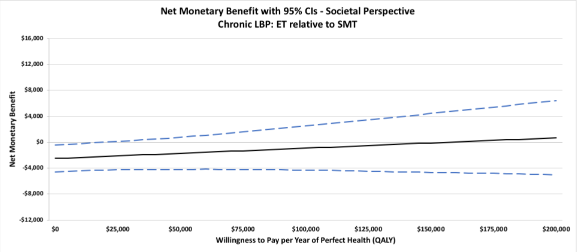

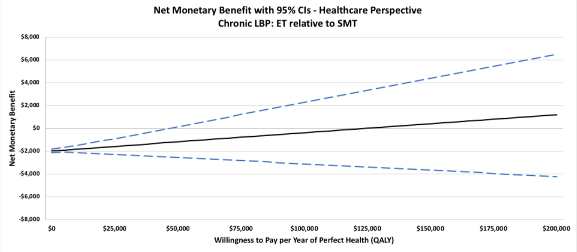

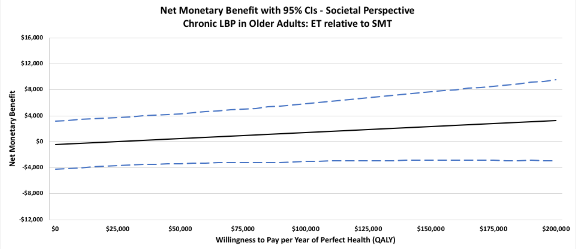

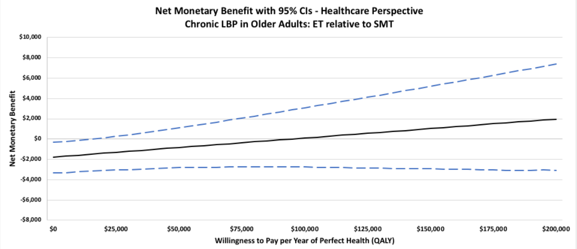

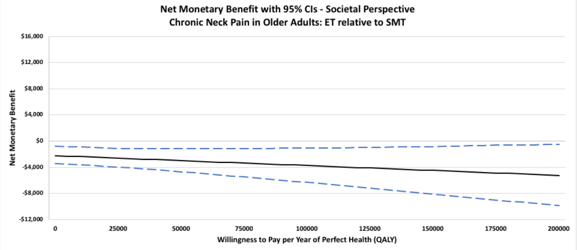

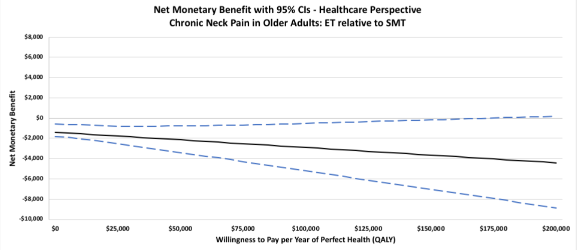

Pain Reduction

Societal Perspective	Healthcare Perspective

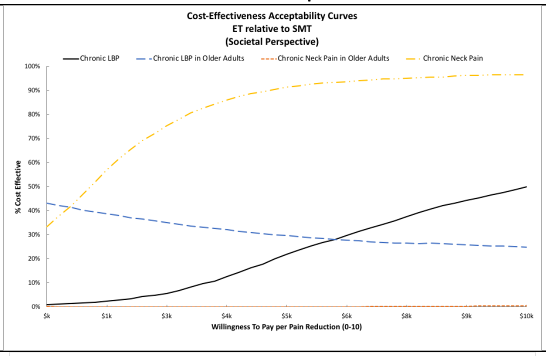

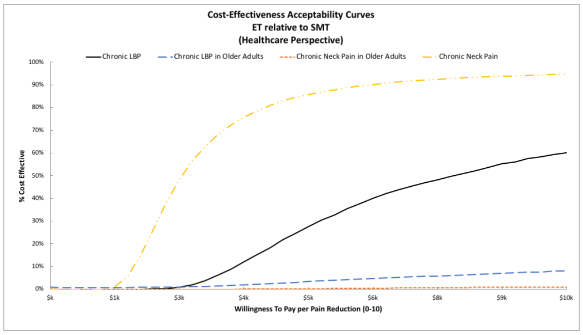

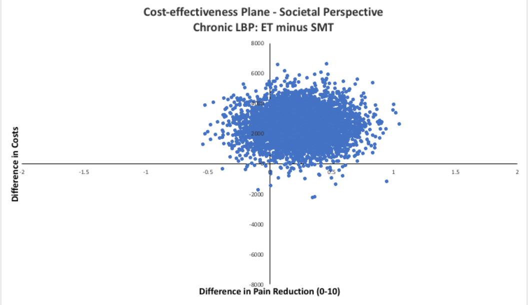

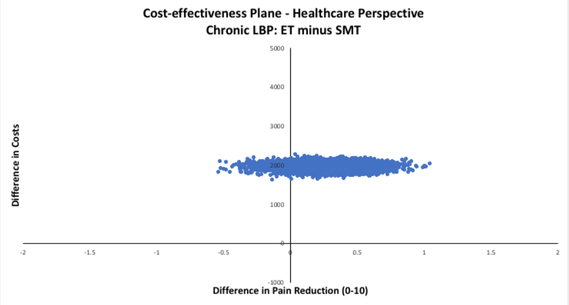

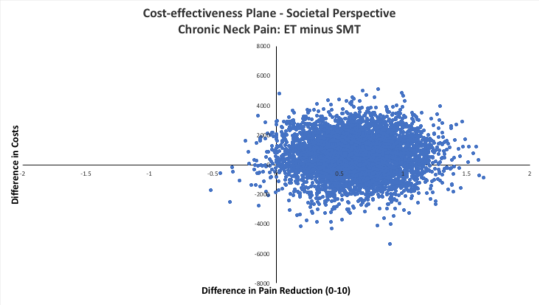

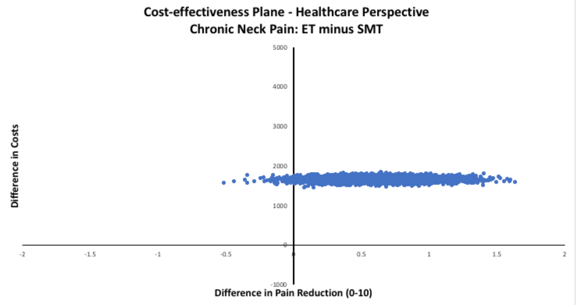

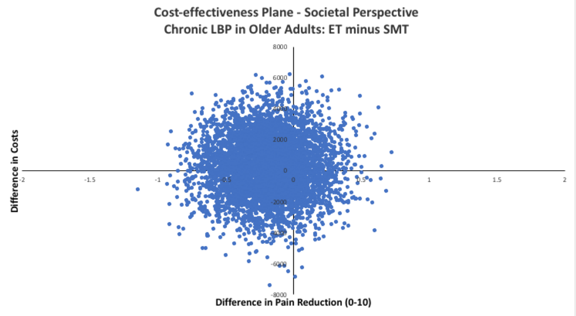

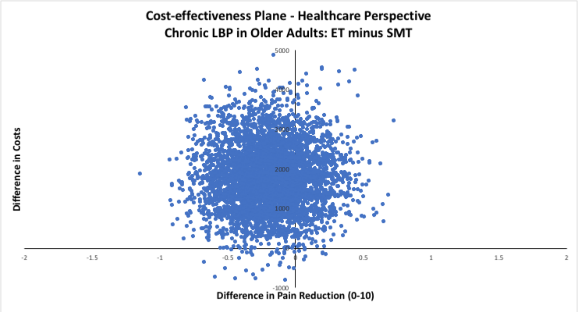

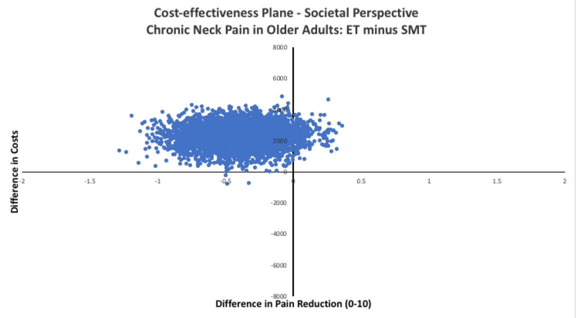

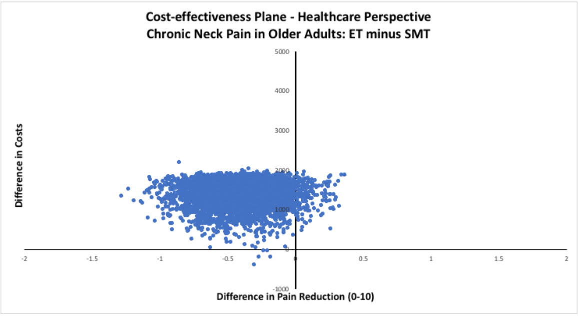

Disability Reduction

Societal Perspective	Healthcare Perspective

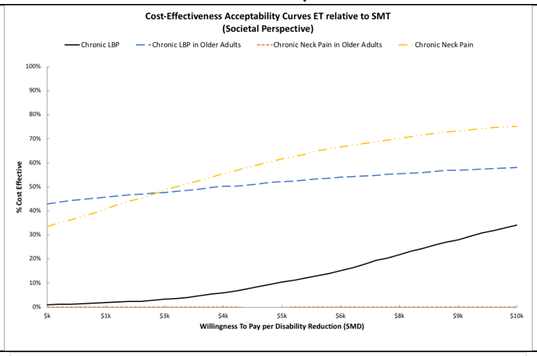

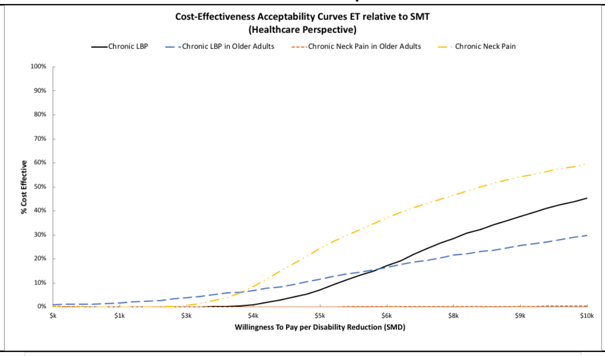

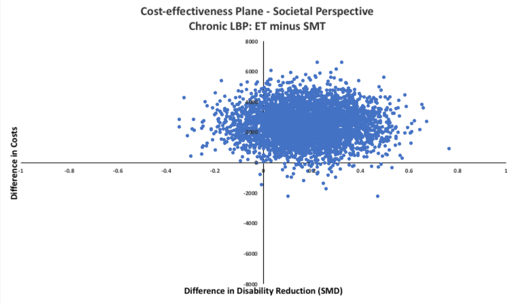

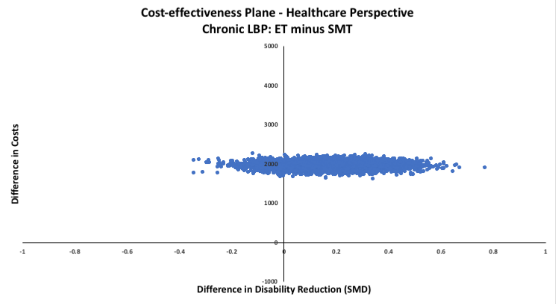

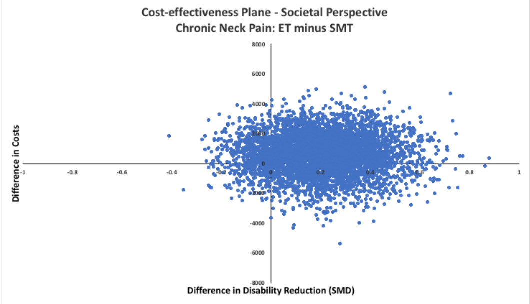

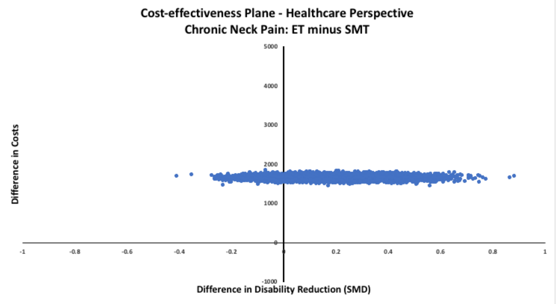

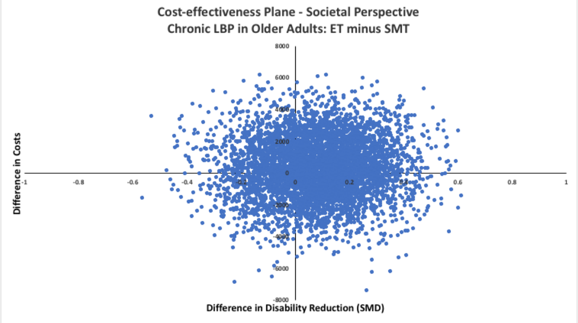

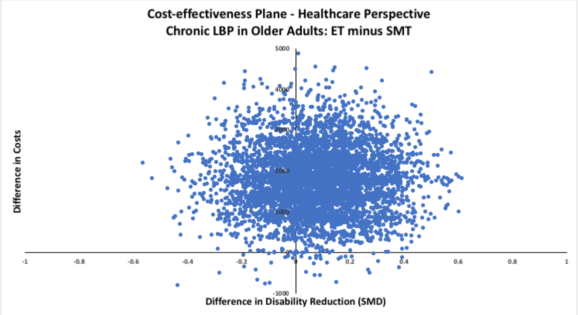

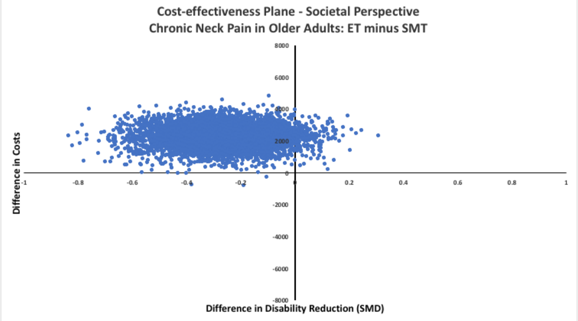

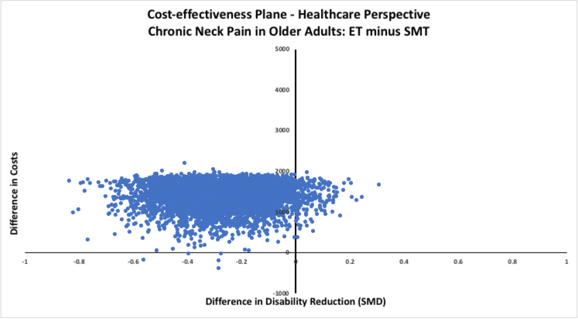

<u>SMT+ET vs ET Figures</u>

QALYs (SF6D)

<table>
<tr><th>Societal Perspective</th><th>Healthcare Perspective</th></tr>
<tr><td>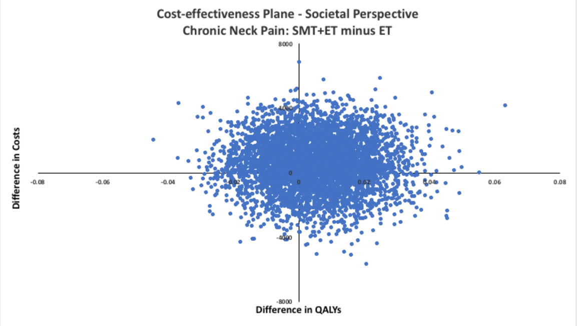
</td><td>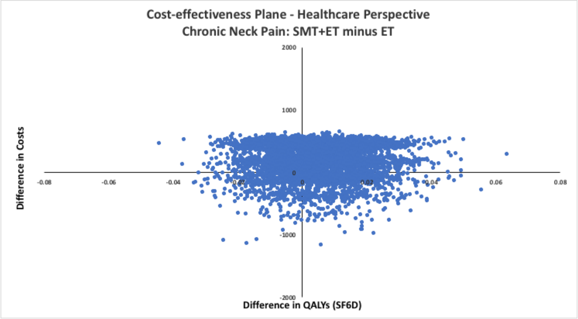
</td></tr>
<tr><td>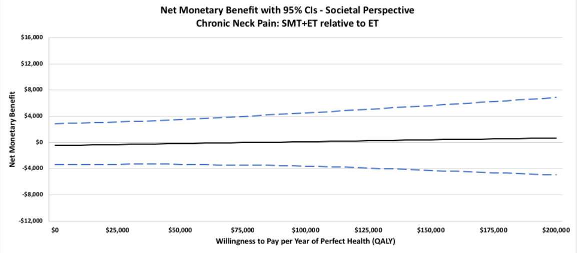
</td><td>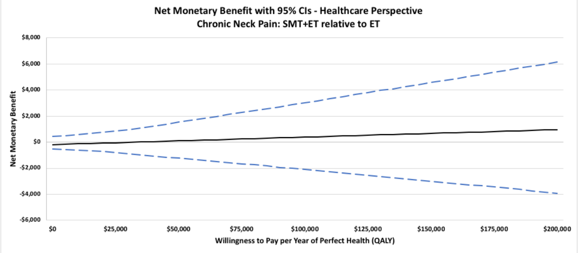
</td></tr>
</table>

QALYs (EQ5D)

Societal Perspective	Healthcare Perspective
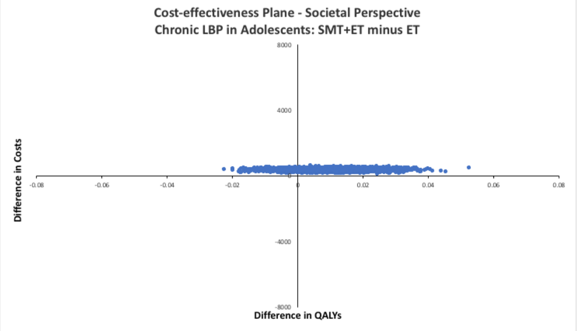	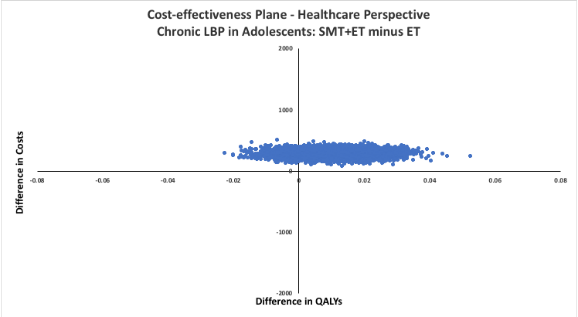
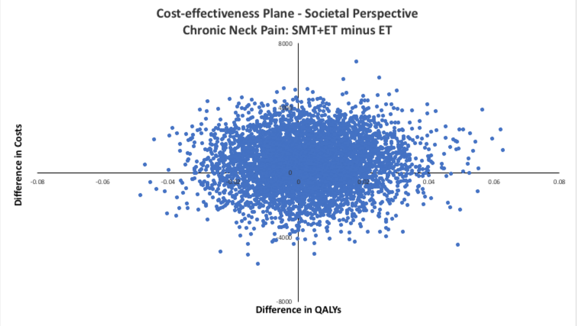	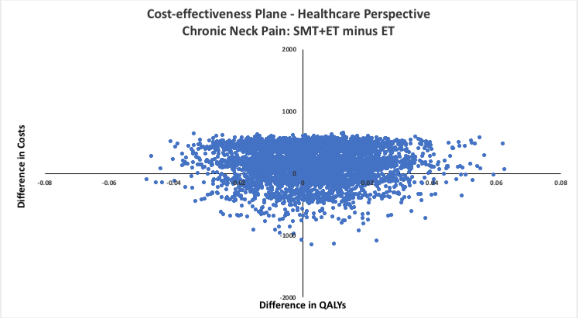
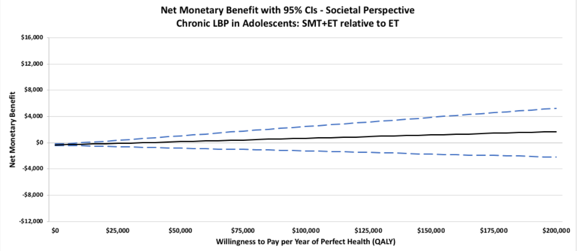	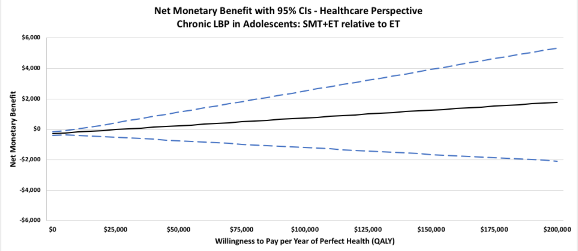
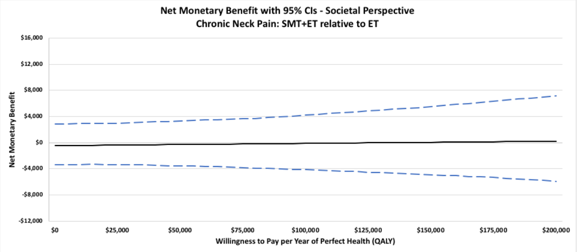	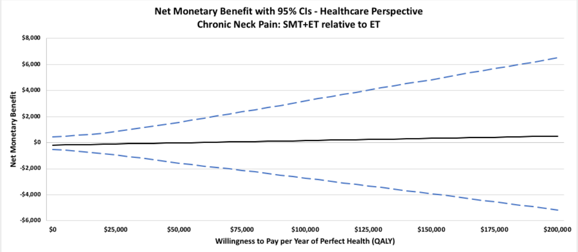

Pain Reduction

Societal Perspective

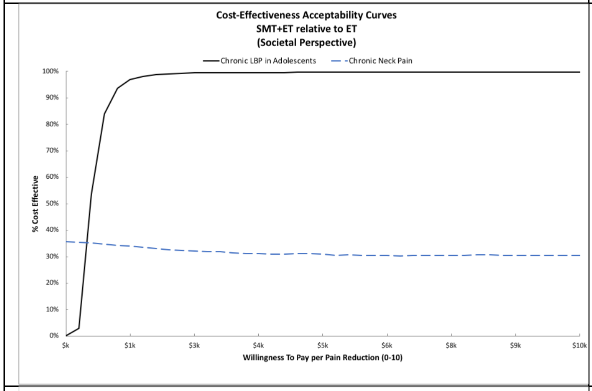

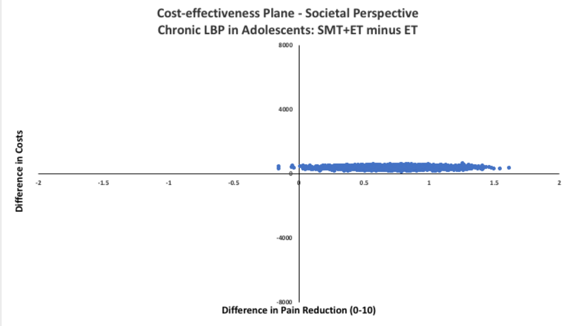

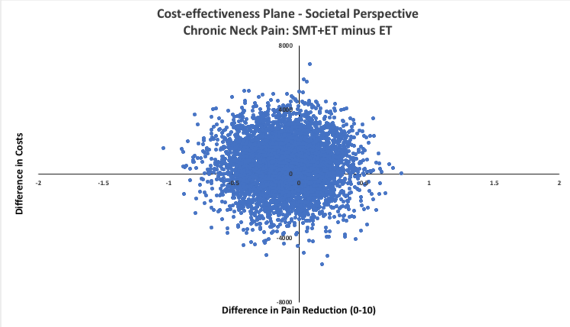

Healthcare Perspective

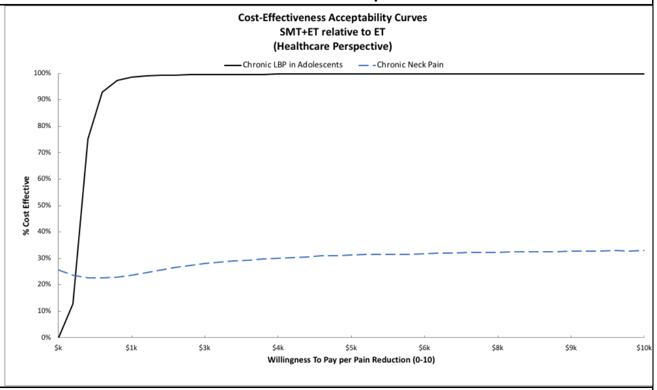

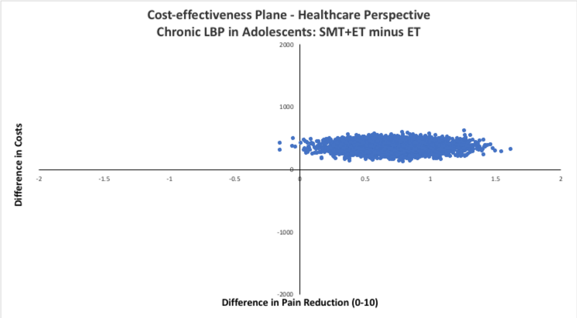

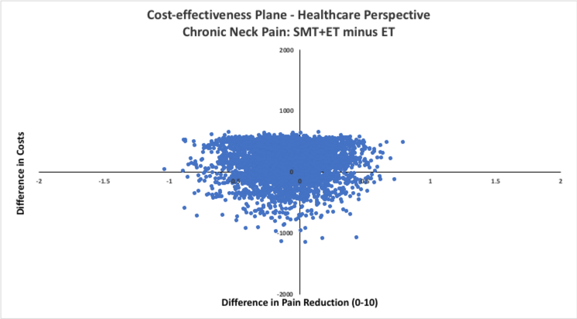

Disability Reduction

<table>
<tr><th>Societal Perspective</th><th>Healthcare Perspective</th></tr>
<tr><td>

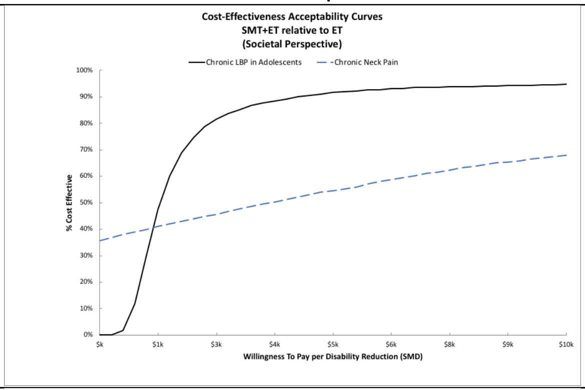

</td><td>

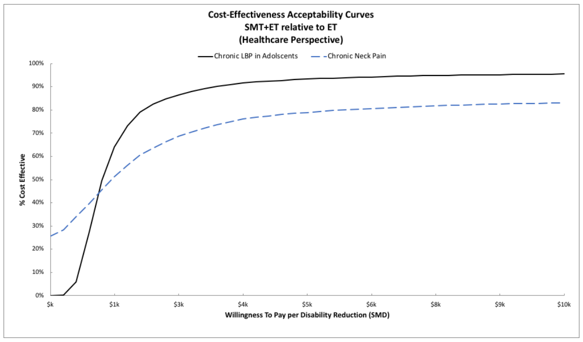

</td></tr>
<tr><td>

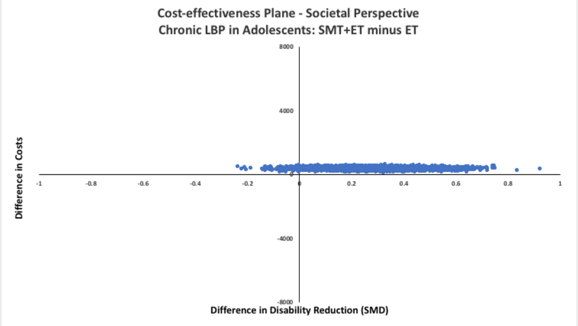

</td><td>

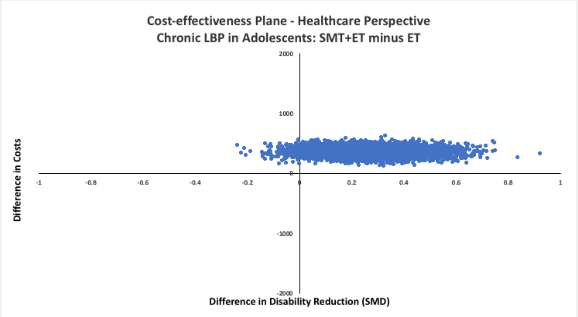

</td></tr>
<tr><td>

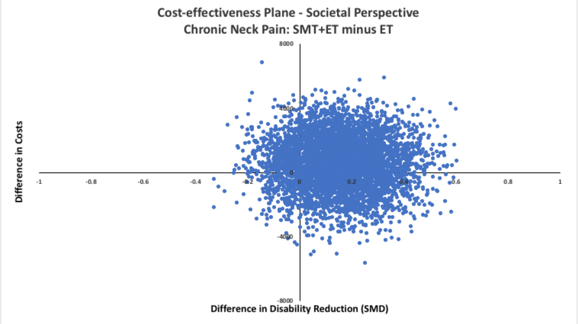

</td><td>

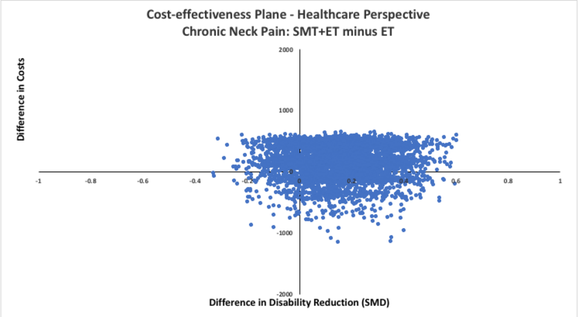

</td></tr>
</table>

ET vs HEA Figures

QALYs (SF6D)

Societal Perspective

Healthcare Perspective

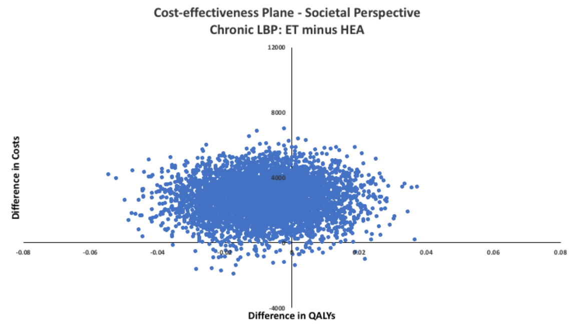

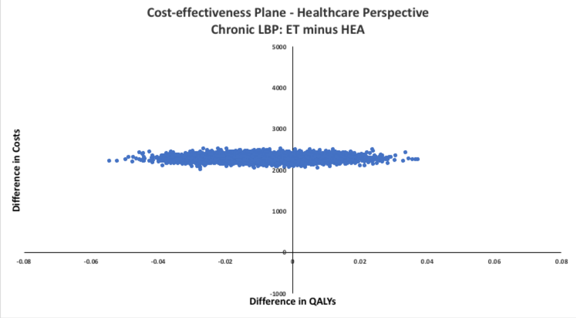

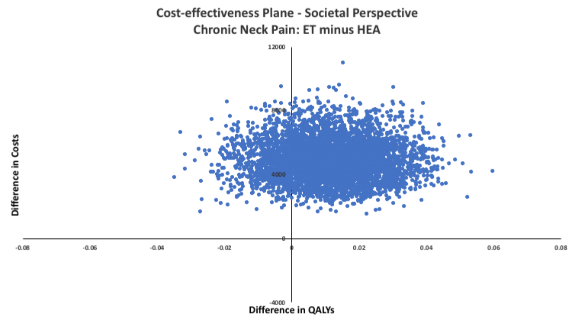

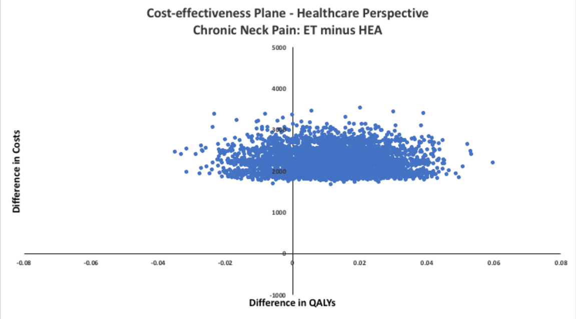

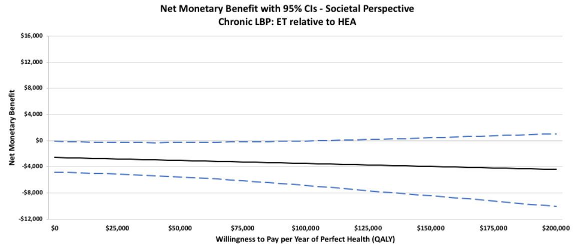

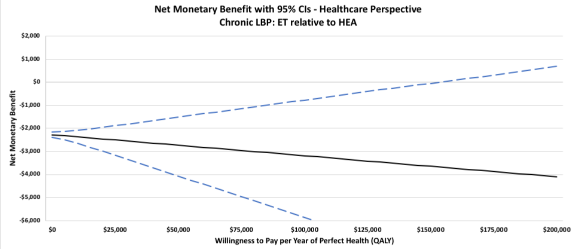

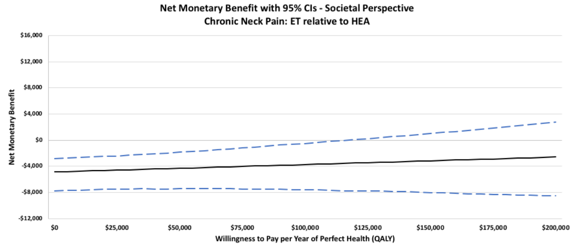

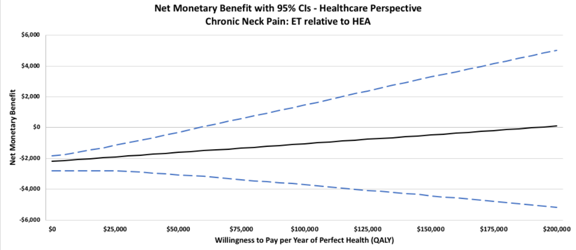

QALYs (EQ5D)

Societal Perspective	Healthcare Perspective
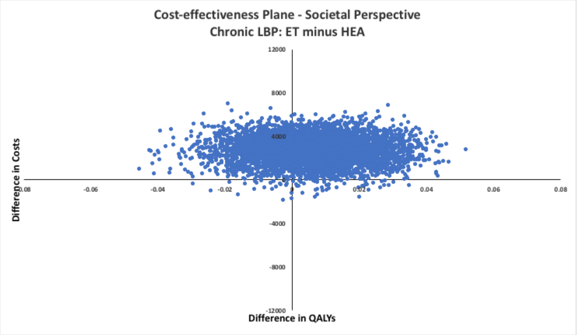	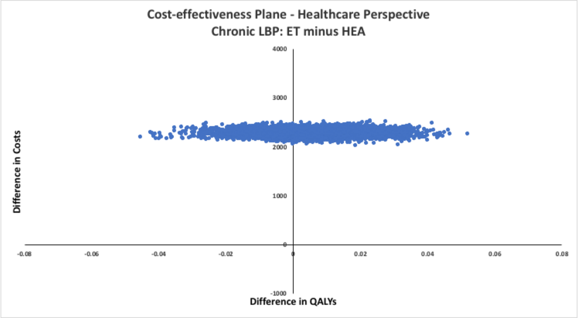
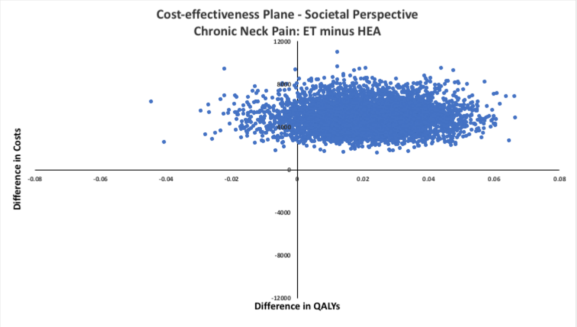	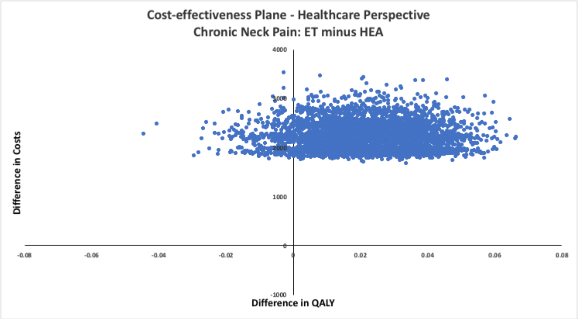
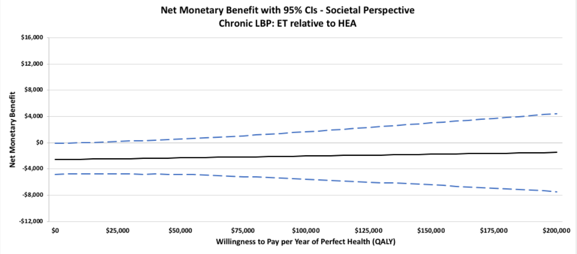	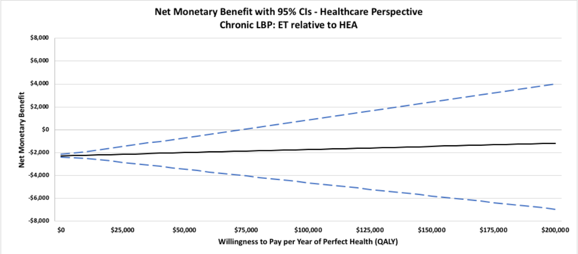
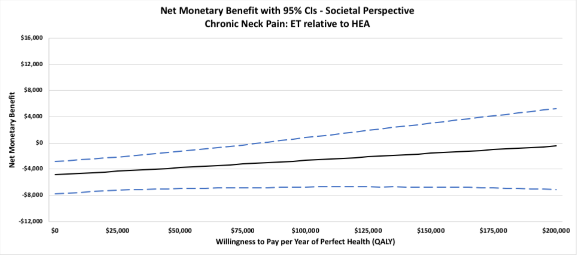	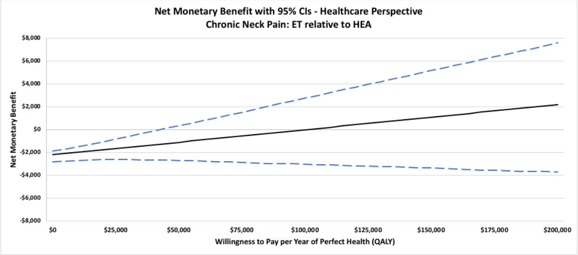

Pain Reduction

Societal Perspective	Healthcare Perspective
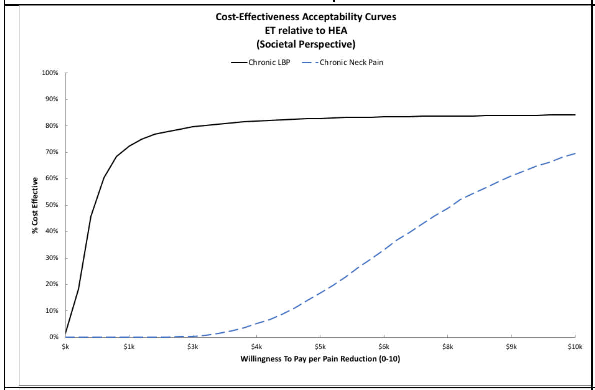	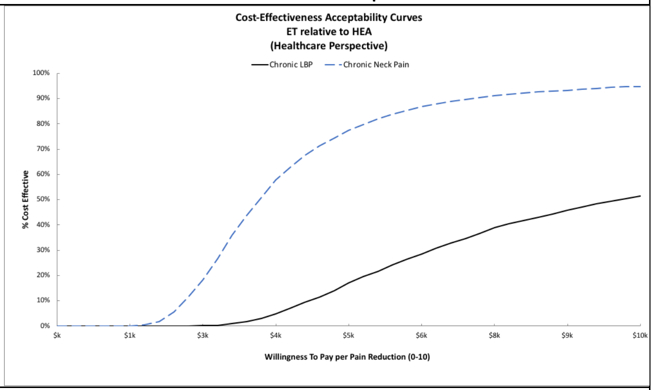
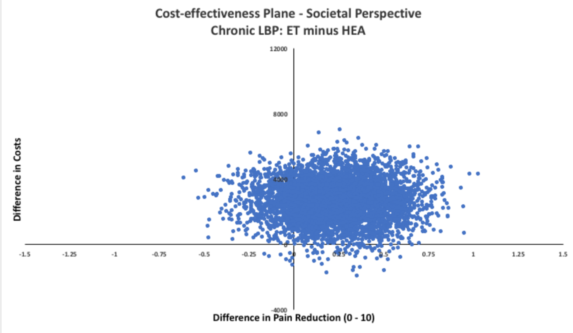	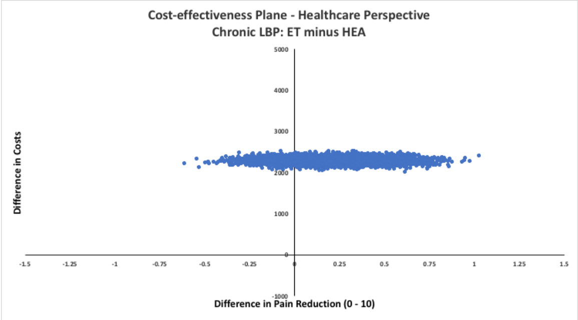
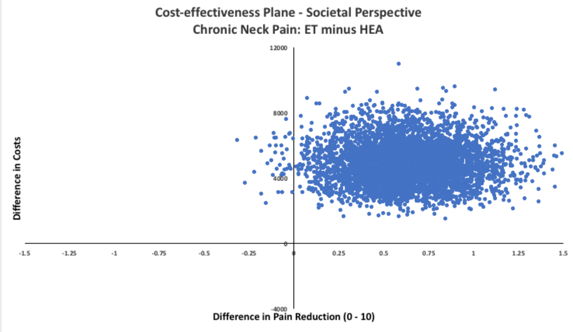	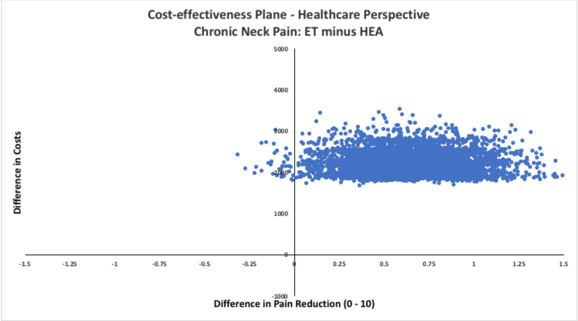

Disability Reduction

Societal Perspective	Healthcare Perspective

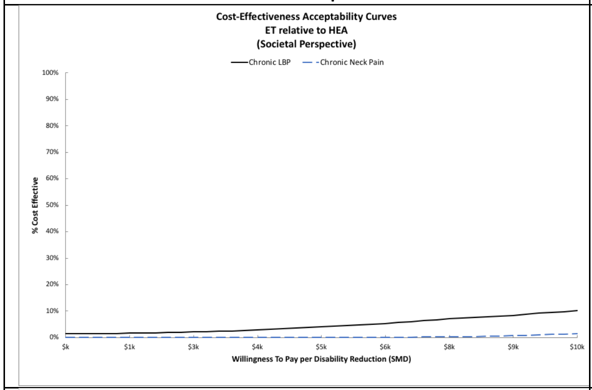

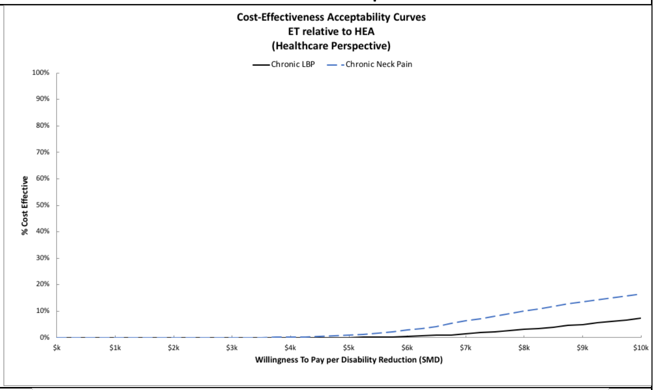

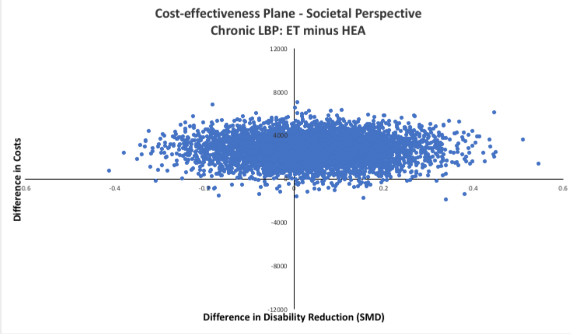

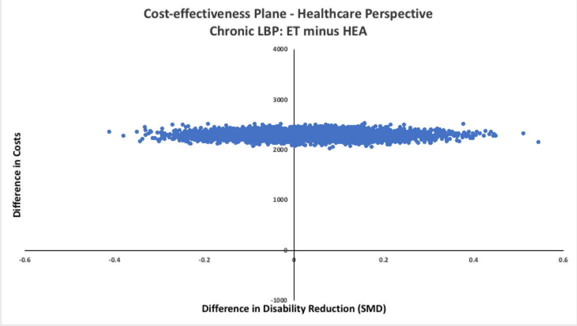

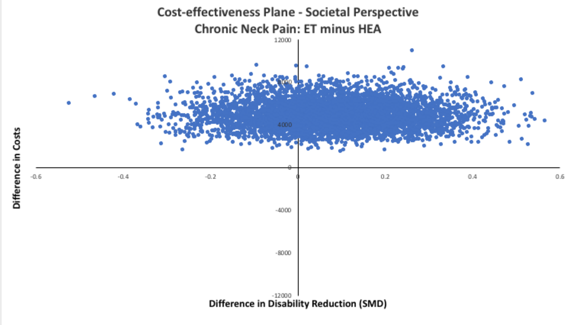

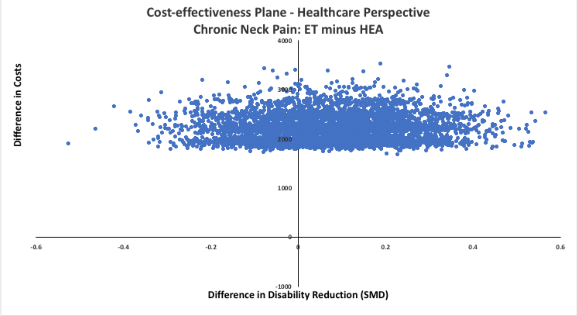

<u>ET+HEA vs HEA Figures</u>

QALYs (SF6D)

Societal Perspective

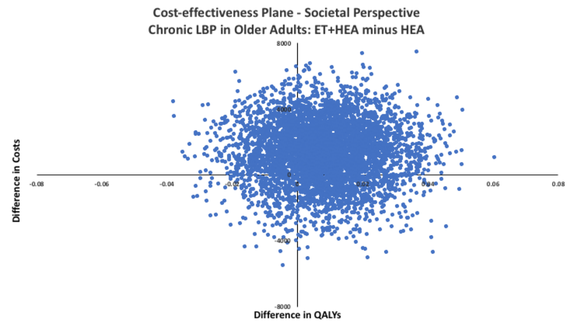

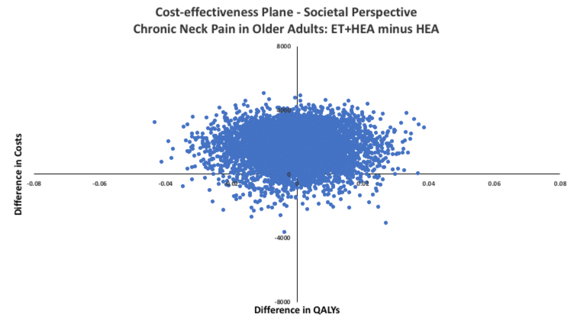

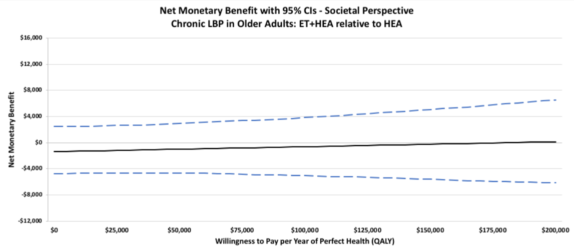

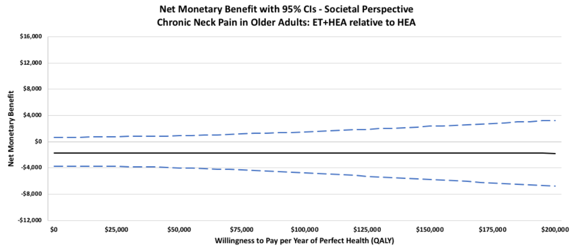

Healthcare Perspective

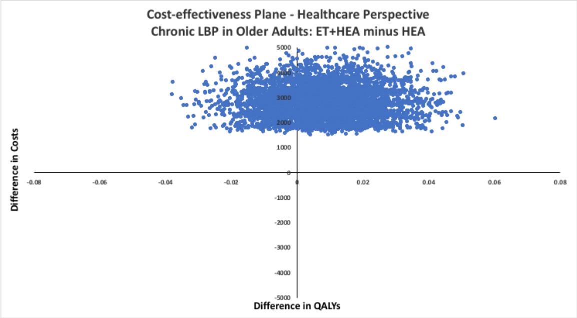

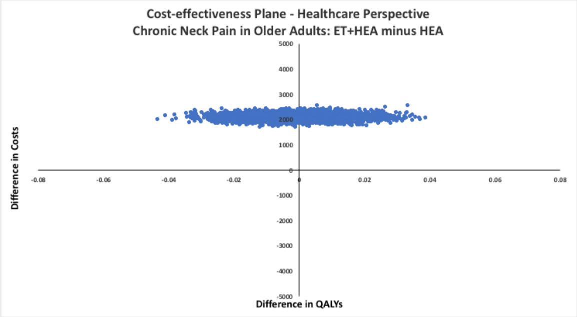

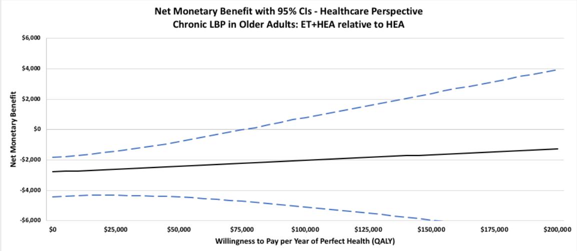

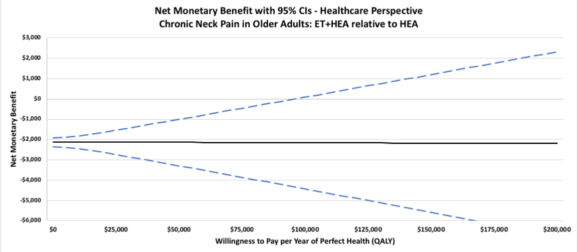

QALYs (EQ5D)

<table>
<tr><th>Societal Perspective</th><th>Healthcare Perspective</th></tr>
<tr><td>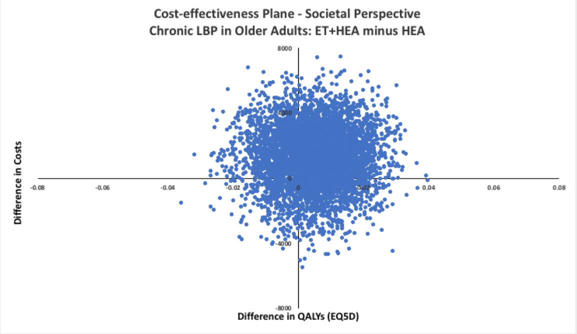
</td><td>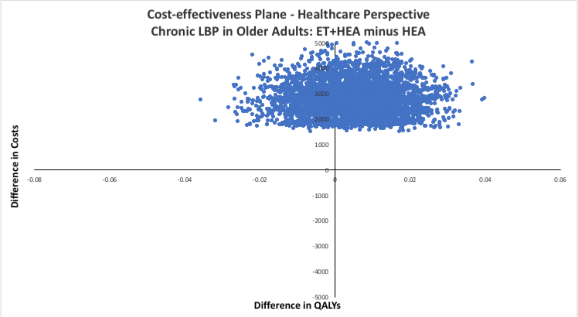
</td></tr>
<tr><td>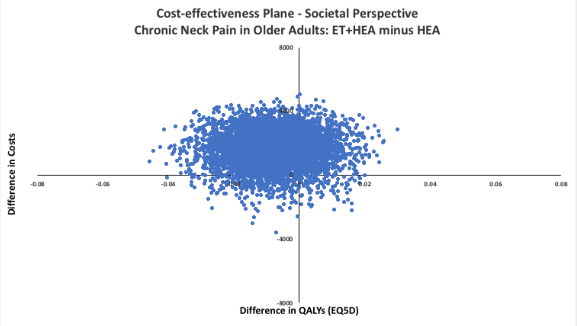
</td><td>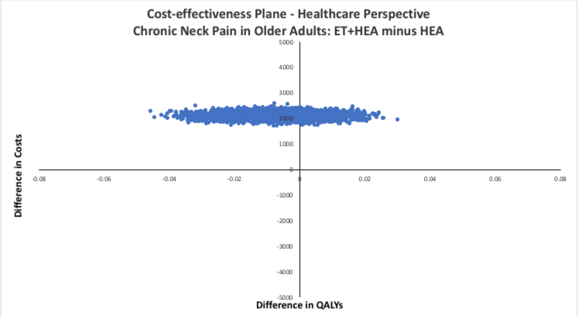
</td></tr>
<tr><td>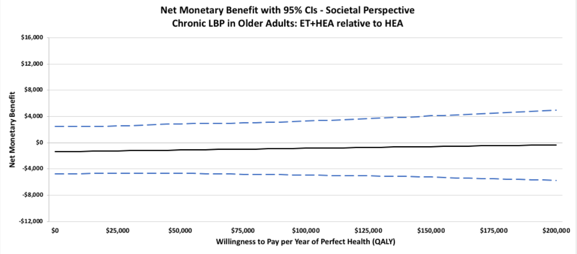
</td><td>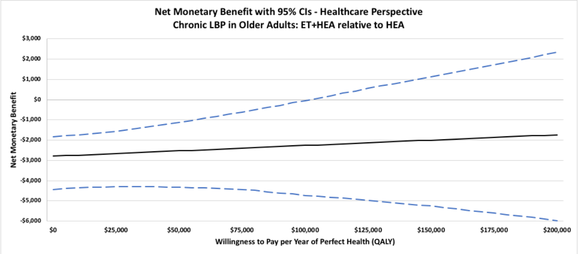
</td></tr>
<tr><td>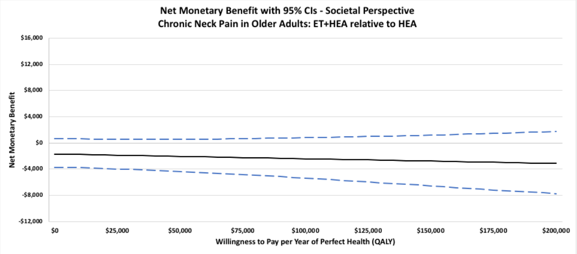
</td><td>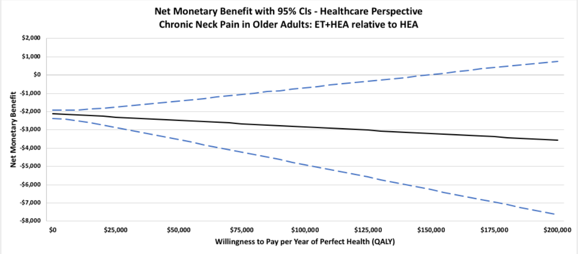
</td></tr>
</table>

Pain Reduction

Societal Perspective	Healthcare Perspective
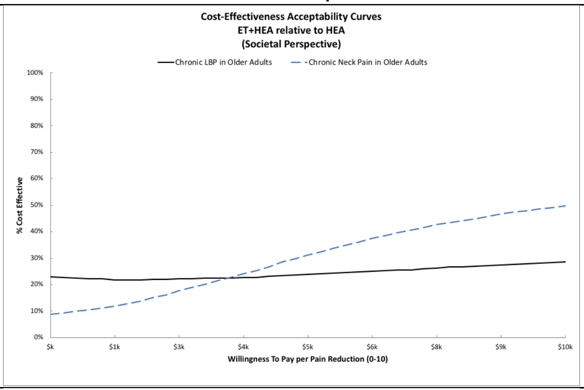	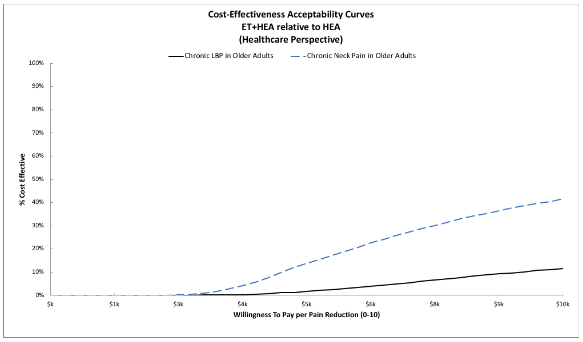
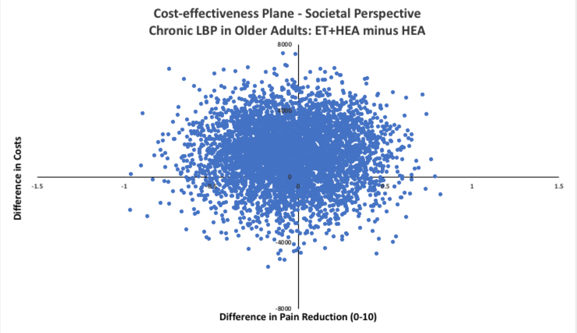	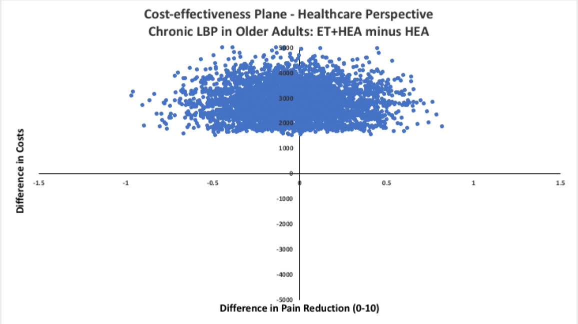
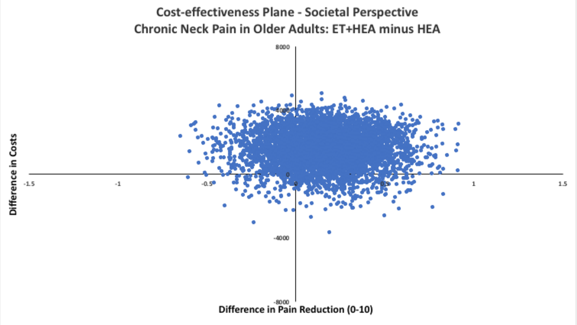	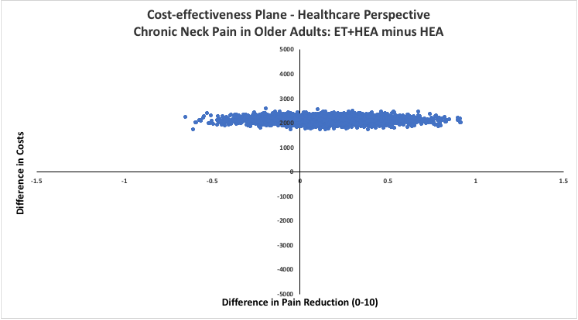

Disability Reduction

Societal Perspective	Healthcare Perspective

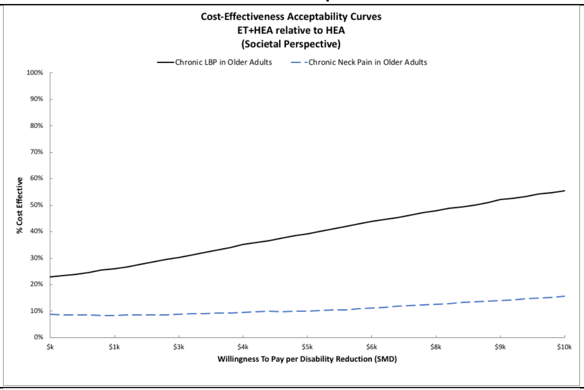

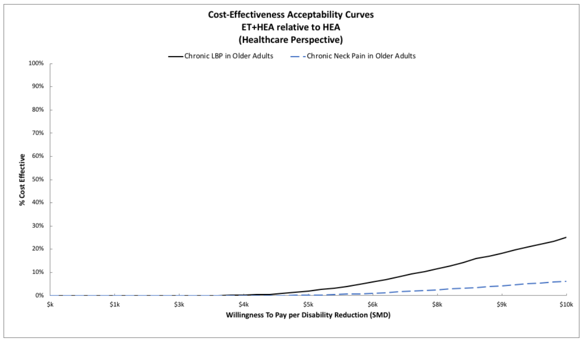

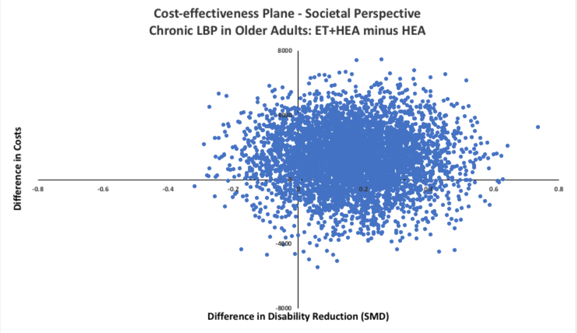

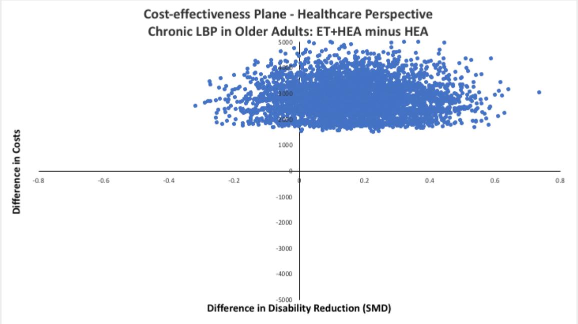

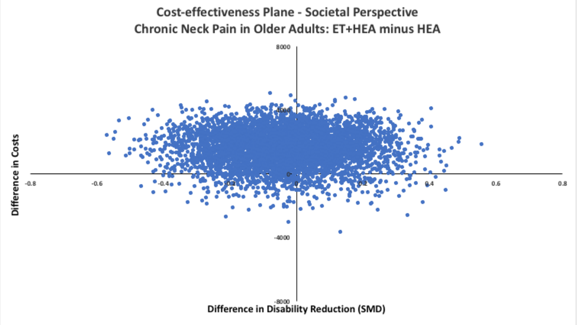

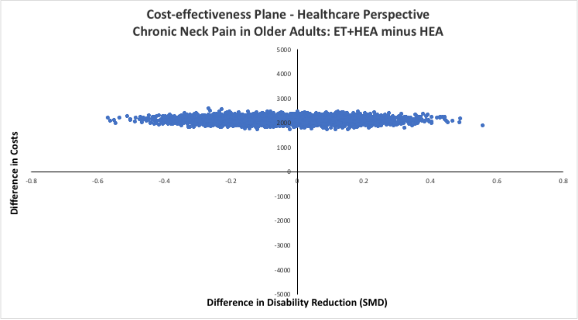

Supplement: Supplementary file 1 — Supplementary Material 1 [file 12998_2025_599_MOESM1_ESM.pdf]
